# Biochemistry and Crystal Structure of Ectoine Synthase: A Metal-Containing Member of the Cupin Superfamily

**DOI:** 10.1371/journal.pone.0151285

**Published:** 2016-03-17

**Authors:** Nils Widderich, Stefanie Kobus, Astrid Höppner, Ramona Riclea, Andreas Seubert, Jeroen S. Dickschat, Johann Heider, Sander H. J. Smits, Erhard Bremer

**Affiliations:** 1 Department of Biology, Laboratory for Microbiology, Philipps-Universität Marburg, Marburg, Germany; 2 X-ray Facility and Crystal Farm, Heinrich-Heine-Universität Düsseldorf, Düsseldorf, Germany; 3 Kekulé-Institute for Organic Chemistry and Biochemistry, Friedrich-Wilhelms-Universität Bonn, Bonn, Germany; 4 Institute of Organic Chemistry, TU Braunschweig, Braunschweig, Germany; 5 Department of Chemistry, Analytical Chemistry, Philipps-Universität Marburg, Marburg, Germany; 6 LOEWE Center for Synthetic Microbiology, Philipps-University Marburg, D-35043 Marburg, Germany; 7 Institute of Biochemistry, Heinrich-Heine-Universität Düsseldorf, Düsseldorf, Germany; Griffith University, AUSTRALIA

## Abstract

Ectoine is a compatible solute and chemical chaperone widely used by members of the *Bacteria* and a few *Archaea* to fend-off the detrimental effects of high external osmolarity on cellular physiology and growth. Ectoine synthase (EctC) catalyzes the last step in ectoine production and mediates the ring closure of the substrate *N*-gamma-acetyl-L-2,4-diaminobutyric acid through a water elimination reaction. However, the crystal structure of ectoine synthase is not known and a clear understanding of how its fold contributes to enzyme activity is thus lacking. Using the ectoine synthase from the cold-adapted marine bacterium *Sphingopyxis alaskensis* (*Sa*), we report here both a detailed biochemical characterization of the EctC enzyme and the high-resolution crystal structure of its apo-form. Structural analysis classified the (*Sa*)EctC protein as a member of the cupin superfamily. EctC forms a dimer with a head-to-tail arrangement, both in solution and in the crystal structure. The interface of the dimer assembly is shaped through backbone-contacts and weak hydrophobic interactions mediated by two beta-sheets within each monomer. We show for the first time that ectoine synthase harbors a catalytically important metal co-factor; metal depletion and reconstitution experiments suggest that EctC is probably an iron-dependent enzyme. We found that EctC not only effectively converts its natural substrate *N*-gamma-acetyl-L-2,4-diaminobutyric acid into ectoine through a cyclocondensation reaction, but that it can also use the isomer *N*-alpha-acetyl-L-2,4-diaminobutyric acid as its substrate, albeit with substantially reduced catalytic efficiency. Structure-guided site-directed mutagenesis experiments targeting amino acid residues that are evolutionarily highly conserved among the extended EctC protein family, including those forming the presumptive iron-binding site, were conducted to functionally analyze the properties of the resulting EctC variants. An assessment of enzyme activity and iron content of these mutants give important clues for understanding the architecture of the active site positioned within the core of the EctC cupin barrel.

## Introduction

Compatible solutes are exploited by members of all three domains of life as versatile cyto-protectants [1], in particular against cellular stress elicited by high osmolarity environments [2–5]. They are especially useful for this latter purpose since their benign nature [6] allows their amassing to exceedingly high cellular concentrations. As a result of compatible solute accumulation, dehydration of the cytoplasm of osmotically stressed cells is counteracted [7], and concomitantly, its solvent properties are optimized for the functioning of vital biochemical and physiological processes [8, 9].

**Fig 1:**
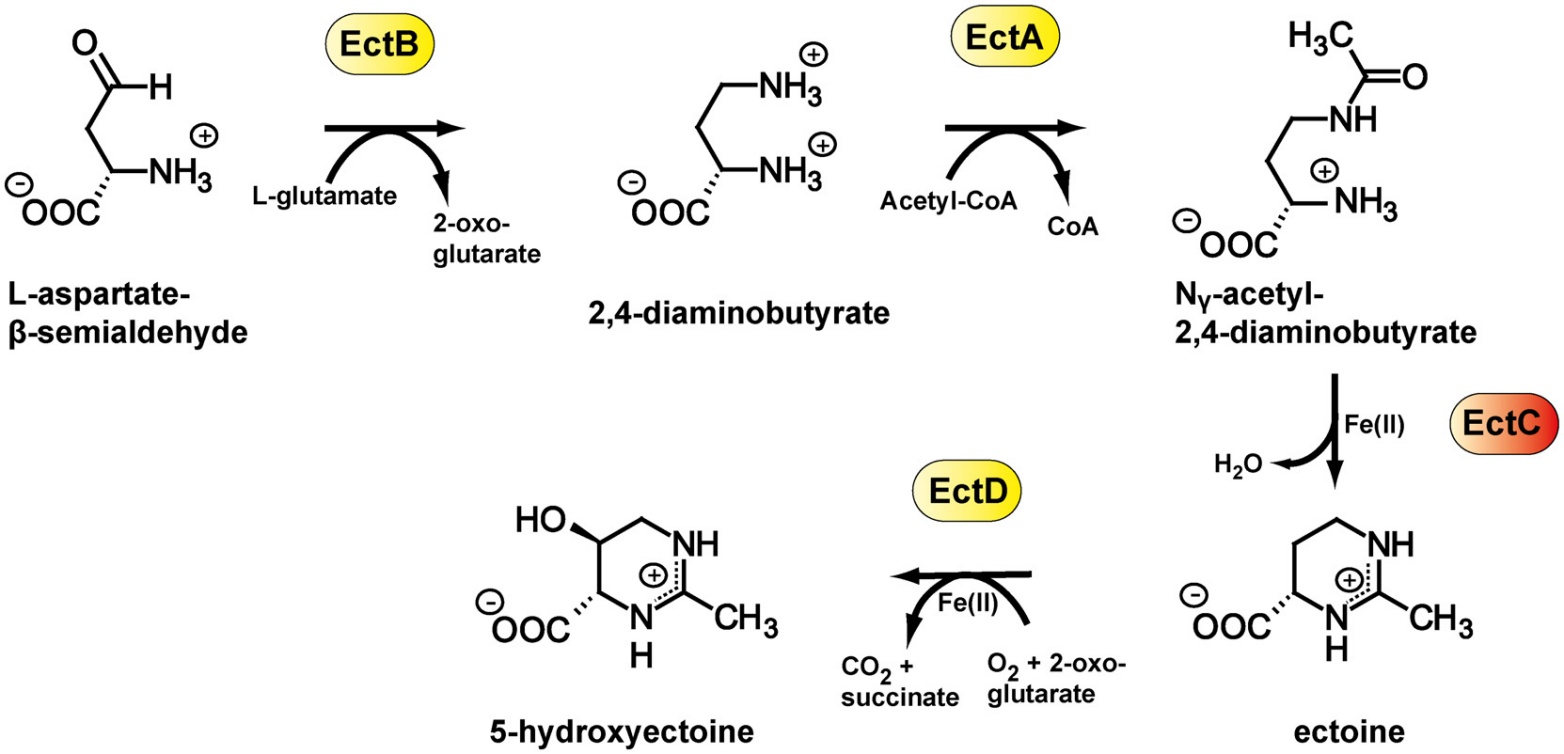
Biosynthetic routes for ectoine and 5-hydroxyectoine. Scheme of the ectoine and 5-hydroxyectoine biosynthetic pathway [20, 21, 25].

Ectoine [(*S*)-2-methyl-1,4,5,6-tetrahydropyrimidine-4-carboxylic acid] [10] and its derivative 5-hydroxyectoine [(4*S*,5*S*)-5-hydroxy-2-methyl-1,4,5,6-tetrahydropyrimidine-4-carboxylic acid] [11] are such compatible solutes [12]. Both marine and terrestrial microorganisms produce them widely [13, 14] in response to osmotic or temperature stress [15–17]. Synthesis of ectoine occurs from the intermediate metabolite L-aspartate-ß-semialdehyde [18, 19] and comprises the sequential activities of three enzymes: L-2,4-diaminobutyrate transaminase (EctB; EC 2.6.1.76), 2,4-diaminobutyrate acetyltransferase (EctA; EC 2.3.1.178), and ectoine synthase (EctC; EC 4.2.1.108) [20, 21] (Fig 1). The ectoine derivative 5-hydroxyectoine, a highly effective stress protectant in its own right [22–24], is synthesized by a substantial subgroup of the ectoine producers [13, 14]. This stereospecific chemical modification of ectoine (Fig 1) is catalyzed by the ectoine hydroxylase (EctD) (EC 1.14.11) [25, 26], a member of the non-heme containing iron(II) and 2-oxoglutarate-dependent dioxygenase superfamily [27]. The remarkable function preserving effects of ectoines for macromolecules and cells [28–31], frequently also addressed as chemical chaperones, led to a substantial interest in exploiting these compounds for biotechnological purposes and medical applications [12, 32, 33].

Here we focus on ectoine synthase (EctC), the key enzyme of the ectoine biosynthetic route [13, 14, 20, 21, 34, 35] (Fig 1). Biochemical characterizations of ectoine synthases from the extremophiles *Halomonas elongata*, *Methylomicrobium alcaliphilum*, and *Acidiphilium cryptum*, and from the nitrifying archaeon *Nitrosopumilus maritimus* have been carried out [14, 35–38]. Each of these enzymes catalyzes as their main activity the cyclization of *N-*γ-acetyl-L-2,4-diaminobutyric acid (*N*-γ-ADABA), the reaction product of the 2,4-diaminobutyrate acetyltransferase (EctA), to ectoine with the concomitant release of a water molecule (Fig 1). In side reactions, EctC can promote the formation of the synthetic compatible solute 5-amino-3,4-dihydro-2H-pyrrole-2-carboxylate (ADPC) through the cyclic condensation of two glutamine molecules [36] and it also possesses a minor hydrolytic activity for ectoine and synthetic ectoine derivatives with either reduced or expanded ring sizes [36, 37].

Although progress has been made with respect to the biochemical characterization of ectoine synthase [14, 35–38], a clear understanding of how its structure contributes to its enzyme activity and reaction mechanism is still lacking. With this in mind, we have biochemically characterized the ectoine synthase from the cold-adapted marine bacterium *Sphingopyxis alaskensis* (*Sa*). We demonstrate here for the first time that the ectoine synthase is a metal-dependent enzyme, with iron as the most likely physiologically relevant co-factor. The EctC protein forms a dimer in solution and our structural analysis identifies it as a member of the cupin superfamily. The two crystal structures that we report here for the (*Sa*)EctC protein (with resolutions of 1.2 Å and 2.0 Å, respectively), and data derived from extensive site-directed mutagenesis experiments targeting evolutionarily highly conserved residues within the extended EctC protein family, provide a first view into the architecture of the catalytic core of the ectoine synthase.

## Materials and Methods

### Chemicals

Ectoine [(*S*)-2-methyl-1,4,5,6-tetrahydropyrimidine-4-carboxylic acid] was a kind gift from bitop AG (Witten, Germany). Anhydrotetracycline (AHT), desthiobiotine and the strepavidin affinity matrix for the purification of *Strep*-tag II labeled proteins was purchased from IBA GmbH (Göttingen, Germany). Hydroxylamine and phenanthroline for the photometric determination of the iron-content of the recombinant (*Sa*)EctC proteins were purchased from Sigma-Aldrich (München, Germany).

### Synthesis of *N*-γ-acetyl-L-2,4-diaminobutyric acid and *N*-α-acetyl-L-2,4-diaminobutyric acid through hydrolysis of ectoine

All chemicals used to synthesize the gamma and alpha forms of *N*-acetyl-l-2,4-diaminobutyric acid (ADABA) for EctC enzyme activity assays were purchased either from Sigma Aldrich (Steinheim, Germany), or Acros (Geel, Belgium). Alkaline hydrolysis of ectoine (284 mg, 2.0 mmol) was accomplished in aqueous KOH (50 mL, 0.1 M) for 20 h at 50°C [39]. The reaction mixture was subsequently neutralized with perchloric acid (60% in water, 4 mL) and the precipitated potassium perchlorate was filtered off. Subsequently, the filtrate was concentrated under reduced pressure. Purification of the residue and separation of the formed compounds was then performed by repeated chromatography on a silica gel column (Merck silica gel 60) using a gradient of ethanol/25% ammonia/water 50:1:2–10:1:2 as eluent to yield pure *N*-γ-ADABA (192 mg, 1.20 mmol, 60%) and *N*-α-ADABA (32 mg, 0.20 mmol, 10%). The identity and purity of theses compounds was unequivocally established by thin-layer chromatography (TLC) and nuclear magnetic resonance (^1^H-NMR and ^13^C-NMR) spectroscopy (S1a and S1b Fig) as described [21, 39] on a Bruker AVIII-400 or DRX-500 NMR spectrometer. (i) Analytical data for *N*-γ-ADABA: TLC: *R*_f_ = 0.55 (ethanol/25% ammonia/water 7:1:2); ^1^H-NMR (400 MHz, D_2_O): *δ* = 3.71 (dd, ^3^*J*(H,H) = 7.6 Hz, ^3^*J*(H,H) = 5.6 Hz, 1H, CH), 3.41–3.24 (m, 2H, CH_2_), 2.15–2.01 (m, 2H, CH_2_), 1.99 (s, 3H, CH_3_) ppm; ^13^C-NMR (100 MHz, D_2_O): *δ* = 177.5 (CO), 177.0 (COOH), 55.3 (CH), 38.3 (CH_2_), 33.0 (CH_2_), 24.6 (CH_3_) ppm. (ii) Analytical data for *N*-α-ADABA: TLC: *R*_f_ = 0.38 (ethanol/25% ammonia/water 7:1:2); ^1^H-NMR (400 MHz, D_2_O): *δ* = 4.24 (dd, ^3^*J*(H,H) = 8.8 Hz, ^3^*J*(H,H) = 5.1 Hz, 1H, CH), 3.07–3.02 (m, 2H, CH_2_), 2.22–2.11 (m, 2H, CH_2_), 2.04 (s, 3H, CH_3_) ppm; ^13^C-NMR (100 MHz, D_2_O): *δ* = 180.1 (CO), 176.7 (COOH), 55.4 (CH), 39.6 (CH_2_), 32.5 (CH_2_), 24.7 (CH_3_) ppm.

### Bacterial strains, plasmids and media

The nucleotide sequence of the *ectC* gene from *S*. *alaskensis* (genome accession number: NC_008048) [40] was used as a template to obtain a codon-optimized *ectC* DNA sequence (Life Technologies, Darmstadt, Germany) for its expression in *E*. *coli*. The nucleotide sequence of the synthetic *ectC* gene was deposited in the NCBI database under accession number KR002036. The synthetic *ectC* gene was used to construct an expression plasmid (pNW12) [41] that is based on the pASG-IBA3 vector (IBA GmbH, Göttingen, Germany). In plasmid pNW12, the *ectC* gene is fused at its 3’ end to a short open reading frame encoding a *Strep*-tag II affinity peptide (NWSHPQFEK). It is transcribed from the TetR-controlled *tet* promoter carried by the backbone of the pASG-IBA3 expression vector. De-repression of *tet* promoter activity can be triggered by adding the synthetic inducer AHT for the TetR repressor to the growth medium. The details of the construction of pNW12 have been reported [41].

Plasmids carrying *ectC* genes were routinely maintained in the *Escherichia coli* strain DH5α (Invitrogen, Karlsruhe, Germany) on LB agar plates containing ampicillin (100 μg ml^-1^). Plasmid DNA was isolated by routine procedures. Minimal medium A (MMA) [42] containing 0.5% (w/v) glucose as the carbon source, 0.5% (w/v) casamino acids, 1 mM MgSO_4_, and 3 mM thiamine was used to cultivate the *E*. *coli* strain BL21 carrying pNW12 for the overproduction of the (*Sa*)EctC protein and its mutant derivatives. No additional metal solution was added to the components of the original recipe of MMA [42].

### Site-directed mutagenesis of the *ectC* gene

Variants of the codon-optimized *ectC* gene from *S*. *alaskensis* present on plasmid pNW12 [41] were prepared by site-directed mutagenesis using the QuikChange Lightning Site-Directed Mutagenesis Kit (Agilent, Waldbronn, Germany) with custom synthesized DNA primers purchased from Microsynth AG (Lindau, Germany). The DNA sequence of the entire coding region of each mutant *ectC* gene was determined by Eurofins MWG (Ebersberg, Germany) to ensure the presence of the desired mutation and the absence of unwanted alterations. Details on the genetic changes introduced into *ectC* genes are listed in Table 1.

**Table 1:** Conversion of N-γ-ADABA into ectoine by (*Sa*)EctC mutant derivatives and their iron-content.

Mutation	Amino acid-substitution	Ectoine formed [mM]	Activity (%)	Iron content of the protein preparation (mol %)
WT	-	9.33 ± 0.28	100	92.1 ± 3.4
TAT/GCT	Tyr-52/Ala	2.53 ± 0.18	27	19.8 ± 1.7
GAA/GCA	Glu-57/Ala	0.97 ± 0.16	10	4.3 ± 2.9
GAA/GAT	Glu-57/Asp	6.19 ± 0.42	66	68.7 ± 5.1
TAT/GCT	Tyr-85/Ala	2.15 ± 0.28	23	8.3 ± 2.3
TAT/TTT	Tyr-85/Phe	2.74± 0.50	29	9.4 ± 3.9
TAT/TGG	Tyr-85/Trp	0.95 ± 0.08	10	5.1 ± 1.7
CAT/GCT	His-93/Ala	0.72 ± 0.09	8	4.5 ± 0.8
CAT/AAT	His-93/Asn	2.14 ± 0.31	23	12.9 ± 2.6
TGG/GCG	Trp-21/Ala	2.41 ± 0.39	26	89.4 ± 4.5
ACG/GCC	Ser-23/Ala	1.98 ± 0.42	21	91.6 ± 2.8
ACC/GCC	Thr-40/Ala	1.12 ± 0.13	12	89.6 ± 2.2
TGT/GCT	Cys-105/Ala	0.96 ± 0.21	10	90.1 ± 1.6
TGT/TCT	Cys-105/Ser	7.81 ± 0.65	84	88.7 ± 3.1
TTT/GCT	Phe-107/Ala	4.77 ± 0.10	51	87.9 ± 2.2
TTT/TAT	Phe-107/Tyr	8.87 ± 0.62	95	90.9 ± 3.9
TTT/TGG	Phe-107/Trp	1.08 ± 0.27	12	72.6 ± 5.8
CAT/GCT	His-117/Ala	4.14 ± 0.27	44	82.9 ± 1.1
CAT/GCT	His-55/Ala	1.53 ± 0.19	16	15.4 ± 4.3
GAA/GCA	Glu-115/Ala	1.92 ± 0.44	21	87.6 ± 4.4
GAA/GAT	Glu-115/Asp	7.15 ± 0.60	77	88.0 ± 3.2
CTG/GCG	Leu-87/Ala	5.81 ± 0.44	62	92.0 ± 1.3
GAT/GCT	Asp-91/Ala	7.48 ± 0.81	80	89.6 ± 2.2
GAT/GAA	Asp-91/Glu	9.13 ± 0.57	98	90.0 ± 1.9
ACC/GCC	Thr-41/Ala	8.84 ± 0.63	95	91.7 ± 1.9
CAT/GCT	His-51/Ala	8.94 ± 0.47	96	90.2 ± 2.6

The conversion of N-γ-ADABA into ectoine by the (*Sa*)EctC protein and its mutant derivatives was monitored in a reaction that contained 10 mM N-γ-ADABA as the substrate, 1 mM FeSO_4_ and 5 μg of the EctC protein under study. The amount of ectoine formed was measured after 20 min of incubation of the enzyme-substrate mixture by HPLC analysis. The iron-content of the investigated protein preparations was determined photometrically [43]; note that in comparison with data obtained via ICP-MS, the colorimetric assay overestimates somewhat the iron content of the (*Sa*)EctC protein preparations.

### Overproduction and purification of recombinant EctC proteins

For the overproduction of the (*Sa*)EctC-*Strep*-tag II protein [41], an overnight culture of strain [BL21 (pNW12)] was prepared in MMA and used to inoculate 1 L of MMA (in a 2 L Erlenmeyer flask) to an OD_578_ of 0.05. The cells were grown on an aerial shaker (set to 180 rpm) at 37°C until the culture reached an OD_578_ of 0.5. At this time point, the growth temperature was lowered to 30°C and the speed of the shaker was reduced to 100 rpm. Growth of the culture was continued and when it reached an OD_578_ of 0.7, AHT was added to the growth medium at a final concentration of 0.2 mg ml^-1^ to boost expression of the recombinant *ectC* gene. After 2 h of further incubation of the culture, the *E*. *coli* cells were harvested by centrifugation and disrupted by passing them several times through a French Pressure cell; a cleared cell lysate was prepared by ultracentrifugation (100 000 g) at 4°C for 1 h [41]. The supernatant of this cleared lysate was then passed through a column filled with 5 ml of Strep-Tactin Superflow material (IBA GmbH, Göttingen, Germany); the column had been equilibrated with a buffer containing 200 mM NaCl and 20 mM Tris-HCl (pH 8). The (*Sa*)EctC-*Strep*-tag II protein was eluted from the affinity matrix with three column volumes of the same buffer containing 2.5 mM desthiobiotin. The recombinant (*Sa*)EctC-*Strep*-tag II protein was then concentrated to either 5 mg ml^-1^ for enzymes assays or 10 mg ml^-1^ for crystallization trials with Vivaspin 6 columns (Satorius Stedim Biotech GmbH, Göttingen, Germany) in the same buffer as described above. Desthiobiotin was not removed by dialysis from these protein preparations. The purified and concentrated (*Sa*)EctC-*Strep*-tag II protein was either used immediately for enzymes assays or kept at 4°C since the flash-freezing of the protein with liquid nitrogen and its subsequent storage at -80°C resulted in a rapid inactivation of ectoine synthase activity. 25 Variants of the (*Sa*)EctC-*Strep*-tag II protein carrying singe amino acid substitutions (Table 1) were overproduced and purified using the same procedure employed for the isolation of the wild-type protein. These mutant proteins behaved like the wild-type (*Sa*)EctC-*Strep*-tag II protein during the overproduction and purification procedure. Protein concentrations were determined both with a Pierce BCA Protein Assay Kit (Thermo Scientific, Schwerte, Germany) using BSA as the standard protein and spectrophotometrically by using an extinction coefficient of 15 470 M^-1^ cm^-1^ for the (*Sa*)EctC-*Strep*-tag II protein at a wavelength of 280 nm. The purity and integrity of the isolated (*Sa*)EctC-*Strep*-tag II proteins was inspected by SDS-polyacrylamide (15%) gel electrophoresis (SDS-PAGE). Molecular mass marker proteins for SDS-PAGE were purchased from LifeTechnologies (Darmstadt, Germany).

### Ectoine synthase enzyme activity assays

The ectoine synthase activity of the (*Sa*)EctC protein was determined by HPLC-based enzyme assays. The initial enzyme activity assays were performed in a 30 μl-reaction volume for 20 min at 20°C. The used standard buffer (20 mM Tris, pH 8.0) contained 150 mM NaCl, 1 mM FeCl_2_, and 10 mM *N*-γ-ADABA. To determine optimal enzyme assay conditions for the (*Sa*)EctC-*Strep*-tag II protein, assay parameters and buffer conditions (e.g., the salt-concentrations, temperature, pH) were individually changed. The finally optimized assay buffer for ectoine synthase activity of the (*Sa*)EctC protein contained 20 mM Tris (pH 8.5), 200 mM NaCl, 1 mM FeCl_2_ and 10 mM *N*-γ-ADABA. Activity assays were run for 20 min at 15°C. Usually, 10 μg of the purified (*Sa*)EctC protein were added to start the enzyme assay. To assess the kinetic parameters of the ectoine synthase, varying concentrations of the substrates were used in the optimized assay buffer with a constant amount (10 μg) of the (*Sa*)EctC protein. The concentration of the natural EctC substrate *N*-γ-ADABA was varied between 0 and 40 mM, whereas that of *N*-α-ADABA was varied between 0 and 200 mM in the enzyme assays. Enzyme reactions were stopped by adding 30 μl of acetonitrile (100%) to the reaction vessel. The samples were centrifuged (13000 rpm, at room temperature for 5 min) to remove denatured proteins; the supernatant was subsequently analyzed for the formation of ectoine by HPLC analysis. Usually, 5- to 10-μl samples were injected into the HPLC system and the reaction product ectoine was analytically detected on a GROM-SIL Amino-1PR column (125 x 4 mm with a particle size of 3μm; purchased from GROM, Rottenburg-Hailfingen, Germany). Synthesis of ectoine by the purified (*Sa*)EctC-*Strep*-tag II protein and its mutant derivatives was monitored using a Infinity 1260 Diode Array Detector (DAD) (Agilent, Waldbronn, Germany) integrated into an Agilent 1260 Infinity LC system (Agilent). The ectoine content of the samples was quantified using the OpenLAB software suite (Agilent). The data shown for each *ectC* mutant (Table 1) were derived from two independent (*Sa*)EctC preparations, and each (*Sa*)EctC protein solution was assayed three times for its enzyme activity.

### Metal depletion and reconstitution of the (*Sa*)EctC protein

To assess the dependency of the ectoine synthase for its enzyme activity on iron and other metals, purified and concentrated (*Sa*)EctC protein preparations (10 μM) were treated with different concentrations of EDTA for 10 minutes. They were subsequently dialyzed to remove the EDTA and the remaining (*Sa*)EctC enzyme activity was analyzed. To determine metal ion specificity of the ectoine synthase, 500 μl of the (*Sa*)EctC protein (100 μM) were initially treated with 1 mM EDTA for 10 minutes to obtain apo-(*Sa*)EctC protein preparations and the EDTA was then removed by dialysis. Enzyme activity assays with 10 μM of such protein preparations were then performed in the presence of either stoichiometric (10 μM) or excess amounts (1 mM) of FeCl_2_, FeCl_3_, ZnCl_2_, CoCl_2_, NiCl_2_, CuCl_2_, and MnCl_2_ to monitor metal ion specificity of the ectoine synthase. Prior to initiation of the enzyme reaction (by addition of the substrate), the (*Sa*)EctC protein solution was incubated with the different indicated metal ions for 10 minutes.

### Determination of the oligomeric state of (*Sa*)EctC protein

To determine the oligomeric state of the (*Sa*)EctC protein in solution, we used high-performance liquid chromatography coupled to multi-angle light scattering detection (HPLC-MALS). A Bio SEC-5 HPLC column (Agilent Technologies Deutschland GmbH, Böblingen, Germany) with a pore size of 300 Å was equilibrated with 20 mM Tris-HCl (pH 7.5), 200 mM NaCl for high-performance liquid chromatography analysis. For these experiments, an Agilent Technologies system connected to a triple-angle light scattering detector (miniDAWN TREOS, Wyatt Technology Europe GmbH, Dernbach, Germany) followed by a differential refractive index detection system (Optilab t-rEX, Wyatt Technology) was used. Typically, 100 μl of purified (*Sa*)EctC protein (2 mg ml^-1^) was loaded onto the Bio SEC-5 HPLC column and the obtained data were analyzed with the ASTRA software package (Wyatt Technology).

### Determination of metal content of recombinant (*Sa*)EctC protein by ICP-MS

The elemental contents of P, Fe, Ni, Cu and Zn of the (*Sa*)EctC-*Strep*-Tag-II protein sample were determined by inductive-coupled plasma mass spectrometry (ICP-MS) using an Agilent 7900 ICP-MS system equipped with a HEN nebulizer and cooled scott spray chamber under standard operating conditions. The isotopes ^31^P, ^56^Fe, ^57^Fe, ^58^Ni, ^60^Ni, ^62^Ni, ^63^Cu, ^65^Cu, ^64^Zn, ^66^Zn, and ^67^Zn were measured under NoGas, He collision and H_2_ reaction mode conditions. Some isotopes are strongly interfered from the matrix (mainly ^56^Fe, ^63^Cu) in the NoGas mode and are therefore rejected. The (*Sa*)EctC protein samples and buffer blanks were diluted 100-fold with ultra pure water and spiked with 10 μg kg^-1^ Y as the internal standard. The calibration of the ICP-MS was performed in the concentration range between 0.1 to 100 μg kg^-1^ using a homemade P standard solution prepared from titrimetrically analyzed H_3_PO_4_ solution and from dilutions of a Merck ICP multi-element standard solution IV (Merck No. 111355, Darmstadt, Germany).

### Photometric determination of non-heme-iron in (*Sa*)EctC and its mutant derivatives

To determine the iron content in our (*Sa*)EctC-*Strep*-Tag-II preparations photometrically [43], 10 nmol of the purified proteins were heated at 80°C for 10 min in 250 μl of a 1% HCl solution. The reaction assay was cooled down on ice and then centrifuged (13000 rpm, 10 min at room temperature). The supernatant was transferred to a new reaction tube, and 750 μl H_2_O, 50 μl of 10% hydroxylamine/HCl, and 250 μl of 0.1% phenanthroline were added to the reaction vessel. After 30 min of incubation at room temperature, the absorbance of the solution was measured at 512 nm. 5 to 40 nmol of ammonium iron(II) sulfate were used for calibration of the assay.

### Crystallization of the (*Sa*)EctC protein

Several conditions under which the (*Sa*)EctC protein formed crystals were found by using commercial screens (Nextal, Qiagen, Hilden, Germany; Molecular Dimensions, Suffolk, UK) in 96-well sitting drop plates (Corning 3553) at 12°C. Homogeneous (*Sa)*EctC protein (0.1 μl from a solution of 11 mg protein ml^-1^) was mixed with 0.1 μl reservoir solution and equilibrated against 50 μl reservoir solution. The most promising condition was found with a solution containing 0.05 M calcium acetate, 0.1 M sodium acetate (pH 4.5), and 40% (v/v) 1,2-propanediol from the Nextal Core IV suite (Qiagen, Hilden, Germany). A second condition under which (*Sa*)EctC crystallized was identified in microbatch setups (1 μl + 1 μl drops) using 20% (w/v) PEG 6000, 0.9 M lithium chloride, and 0.1 M citric acid (pH 5) from the Nextal Core II suite (Qiagen, Hilden, Germany). These conditions were optimized by grid screens around the initial condition and/or after the addition of *tert*-butanol as an additive. Large crystals were obtained either without any additive or after the addition of *tert*-butanol to the (*Sa*)EctC protein solution 30 minutes before the drops were spotted. Crystals reached their maximum dimensions of about 50 × 50 × 70 μm^3^ after 3–10 weeks. The crystals were fished after overlaying the drop with 2 μl mineral oil and flash frozen in liquid nitrogen. To obtain heavy atom derivatized crystals, methylmercury(II) chloride was added (final concentration: 0.5 mM) to the crystals in their drop for 30 minutes before they were fished and flash frozen in liquid nitrogen.

### Data processing and structure determination

Native data sets were collected from a single crystal of (*Sa*)EctC obtained from the various crystallization trials at the ERSF beamline ID23eh2 (Grenoble, France) at 100 K. These data sets were processed using the XDS package [44] and scaled with XSCALE [45] showing a maximum resolution of 1.2 Å. To obtain initial phases of (*Sa*)EctC, a mercury-derivatized crystal was used to collect a conservative dataset at 2.8 Å resolution. The data were processed and scaled as described above, before the program AUTORICKSHAW using single isomorphous replacement (SIRAS) [46], was used to localize the Hg atom, phase and built an initial model of the (*Sa*)EctC protein. This initial model was used as a template for molecular replacement on the 2.0 Å dataset revealing four monomers in the asymmetric unit. Once the 2.0 Å structure was refined, a single monomer of this structure was used as a template for molecular replacement to phase the 1.2 Å resolution dataset using the PHENIX software [47]. Model building and refinement were performed using COOT [48], Refmac5 [49] and Phenix_refine [47]. Data refinement statistics and model content are summarized in Table 1.

### PDB accession numbers

The atomic coordinates and structural factors have been deposited into the Protein Data Bank (PDB) (Brookhaven, USA) under the following accession codes: 5BXX (for the “semi-closed” (*Sa*)EctC structure) and 5BY5 (for the “open” (*Sa*)EctC structure).

### Figure preparation of crystal structures

Figures of the crystal structures of *Sa*EctC were prepared using the PyMol software suite (www.pymol.org) [50].

## Results

### Overproduction, purification and oligomeric state of the ectoine synthase in solution

We focused our biochemical and structural studies on the ectoine synthase from *S*. *alaskensis* [(*Sa*)EctC], a cold-adapted marine ultra-microbacterium [40], from which we recently also determined the crystal structure of the ectoine hydroxylase (EctD) in complex with either its substrate or its reaction product [27]. We expressed a codon-optimized version of the *S*. *alaskensis ectC* gene in *E*. *coli* to produce a recombinant protein with a carboxy-terminally attached *Strep*-tag II affinity peptide to allow purification of the (*Sa*)EctC-*Strep*-Tag-II protein by affinity chromatography. The (*Sa*)EctC protein was overproduced and isolated with good yields (30–40 mg L^-1^ of culture) and purity (S2a Fig). Conventional size-exclusion chromatography (SEC) has already shown that (*Sa*)EctC preparations produced in this fashion are homogeneous and that the protein forms dimers in solution [41]. High performance liquid chromatography coupled with multi-angle light-scattering detection (HPLC-MALS) experiments carried out here confirmed that the purified (*Sa*)EctC protein was mono-disperse and possessed a molecular mass of 33.0 ± 2.3 kDa (S2b Fig). This value corresponds very well with the theoretically calculated molecular mass of an (*Sa*)EctC dimer (molecular mass of the monomer, including the *Strep*-tag II affinity peptide: 16.3 kDa). Such a quaternary assembly as dimer has also been reported for the EctC proteins from *H*. *elongata* and *N*. *maritimus* [14, 35].

### Biochemical properties of the ectoine synthase

The EctA-produced substrate of the ectoine synthase, *N-*γ-acetyl-L-2,4-diaminobutyric acid (*N*-γ-ADABA) (Fig 1), is commercially not available. We used alkaline hydrolysis of ectoine [39] and subsequent chromatography on silica gel columns to obtain *N*-γ-ADABA in chemically highly purified form (S1a Fig). This procedure also yielded the isomer of *N*-γ-ADABA, *N*-α-acetyl-L-2,4-diaminobutyric acid (*N*-α-ADABA) (S1b Fig) [39]. *N*-α-ADABA has so far not been considered as a substrate for EctC, but microorganisms that use ectoine as a nutrient produce it as an intermediate during catabolism [51].

Using *N*-γ-ADABA as the substrate, we initially evaluated a set of biochemical parameters of the recombinant (*Sa*)EctC protein. *S*. *alaskensis*, from which the studied ectoine synthase was originally derived, is a microorganism that is well-adapted to a life in permanently cold ocean waters [40]. Consistent with the physicochemical attributes of this habitat, the (*Sa*)EctC protein was already enzymatically active at 5°C, had a temperature optimum of 15°C and was able to function over a broad range of temperatures (S3a Fig). It possessed an alkaline pH optimum of 8.5 (S3b Fig), a value similar to the ectoine synthases from the halo-tolerant *H*. *elongata* (pH optimum of 8.5 to 9.0) [35], the alkaliphile *M*. *alcaliphilum* (pH optimum of 9.0) [38], and the acidophile *Acidiphilium cryptum* (pH optimum of 8.5 to 9.0) [37], whereas the EctC protein from *N*. *maritimus* has a neutral pH optimum (pH 7.0) [14].

The salinity of the assay buffer had a significant influence on the maximal enzyme activity of the (*Sa*)EctC protein. An increase in either the NaCl or the KCl concentration led to an approximately 5-fold enhancement of the ectoine synthase activity. The maximum enzyme activity of (*Sa*)EctC occurred around 250 mM NaCl or KCl, respectively. (*Sa*)EctC is a highly salt-tolerant enzyme since it exhibited substantial enzyme activity even at NaCl and KCl concentrations of 1 M in the assay buffer (S3c and S3d Fig). The stimulation of EctC enzyme activity by salts has previously also been observed for other ectoine synthases [14, 35, 37, 38].

### The ectoine synthase is a metal-containing protein

**Fig 2:**
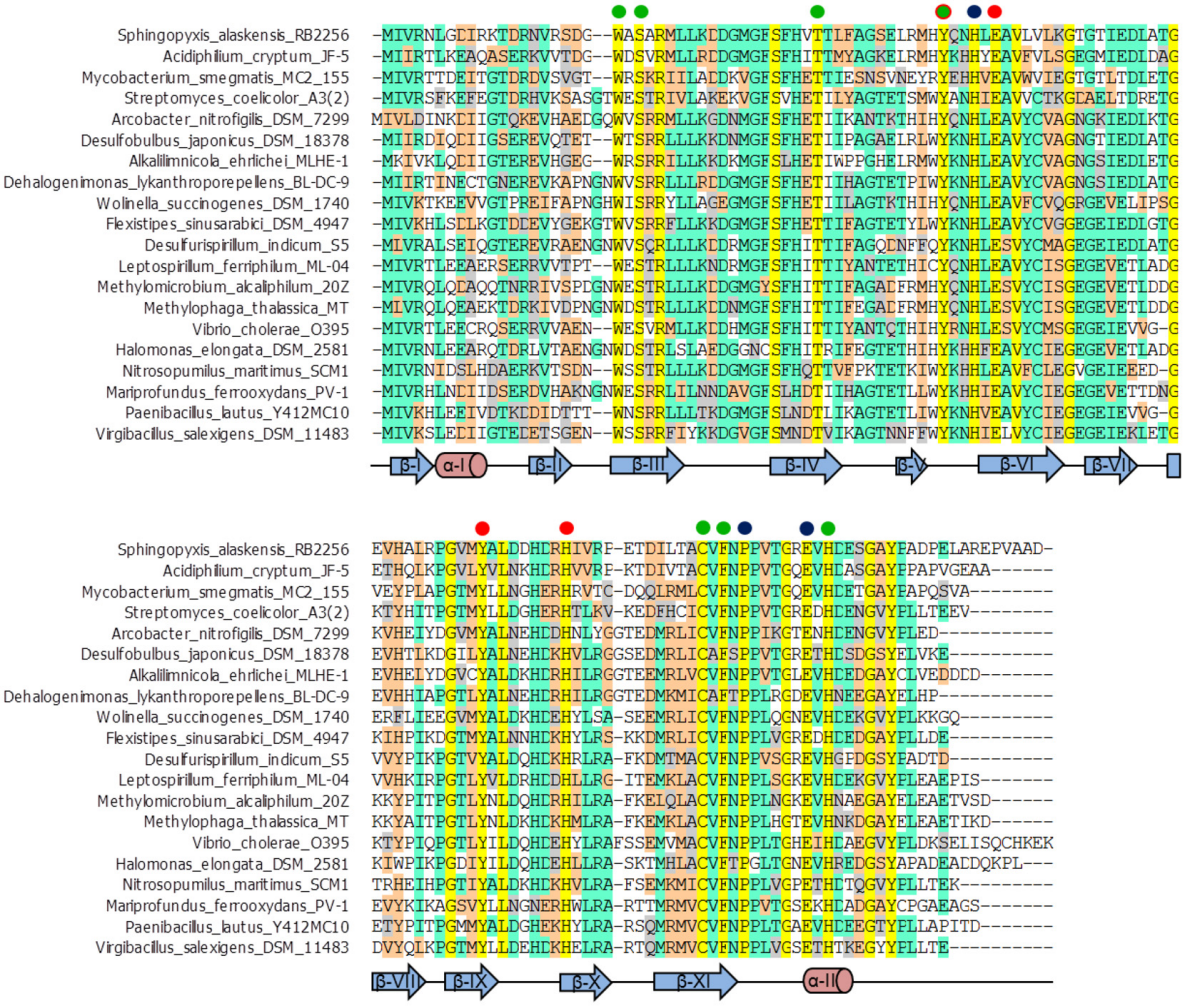
Abbreviated alignment of EctC-type proteins. The amino acid sequences of 20 selected EctC-type proteins are compared. Strictly conserved amino acid residues are shown in yellow. Dots shown above the (*Sa*)EctC protein sequence indicate residues likely to be involved in iron-binding (red), ligand-binding (green) and stabilization of the loop-architecture (blue). The conserved residue Tyr-52 with so-far undefined functions is indicated by a green dot circled in red. Secondary structural elements (α-helices and β-sheets) found in the (*Sa*)EctC crystal structure are projected onto the amino acid sequences of EctC-type proteins.

Considerations based on bioinformatics [52] suggests that EctC belongs to the cupin superfamily [52–55]. Most of these proteins contain catalytically important transition state metals such as iron, copper, zinc, manganese, cobalt, or nickel [52–55]. Cupins contain two conserved motifs: G(X)_5_**H**X**H**(X)_3,4_**E**(X)_6_G and G(X)_5_PXG(X)_2_**H**(X)_3_N (the letters in bold represent those residues that often coordinate the metal). Inspection of a previous alignment of the amino acid sequences of 440 EctC-type proteins [13] revealed that the canonical metal-binding motif(s) of cupin-type proteins [53–55] is not conserved among members of the extended ectoine synthase protein family [13, 14]. An abbreviated alignment of the amino acid sequence of EctC-type proteins is shown in Fig 2.

Since variations of the above-described metal-binding motif occur frequently [52–54], we experimentally investigated the presence and nature of the metal that might be contained in the (*Sa*)EctC protein by inductive-coupled plasma mass spectrometry (ICP-MS). For this analysis we used recombinant (*Sa*)EctC preparations from three independent protein overproduction and purification experiments. The ICP-MS analyses yielded an iron content of 0.66 ± 0.06 mol iron per mol of protein and the used (*Sa*)EctC protein preparations also contained a minor amount of zinc (0.08 mol zinc per mol of protein). All other assayed metals (copper and nickel) were only present in trace amounts (0.01 mol metal per mol of protein, respectively). The presence of iron in these (*Sa*)EctC protein preparations was further confirmed by a colorimetric method that is based on an iron-complexing reagent [43]; this procedure yielded an iron-content of 0.84 ± 0.05 mol per mol of (*Sa*)EctC protein. Hence, both ICP-MS and the colorimetric method clearly established that the recombinantly produced ectoine synthase from *S*. *alaskensis* is an iron-containing protein. We note in this context, that the values obtained for the iron content of the (*Sa*)EctC proteins varied by approximately 10 to 20% between the two methods. The reason for this difference is not known, but indicates that the well established colorimetric assay [43] probably overestimates the iron content of (*Sa*)EctC protein preparations to a certain degree.

### A metal cofactor is important for the catalytic activity of EctC

**Fig 3:**
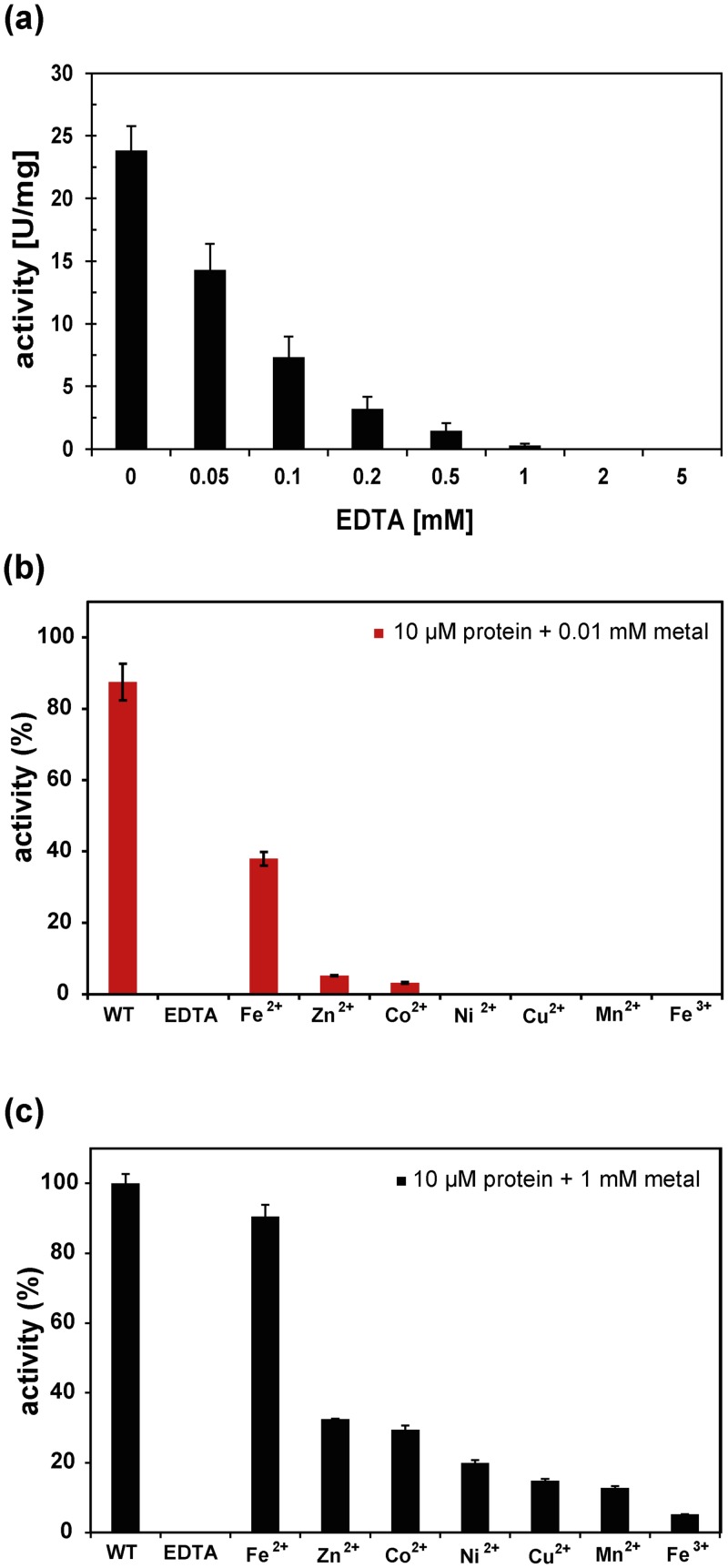
Dependency of the ectoine synthase activity on metals. (a) Impact of the iron-chelator EDTA on the enzyme activity of the purified (*Sa*)EctC protein. Metal depletion and reconstitution experiments with (b) stoichiometric and (c) excess amounts of metals. The (*Sa*)EctC protein was present at a concentration of 10 μM. The level of enzyme activity given in (b) is benchmarked relative to that of ectoine synthase enzyme assays in which 1 mM FeCl_2_ was added.

The iron detected in the (*Sa*)EctC protein preparations could serve a structural role, or most likely, could be critical for enzyme catalysis as is the case for many members of the cupin superfamily [52–55]. To address these questions, we incubated the (*Sa*)EctC enzyme with increasing concentrations of the metal chelator ethylene-diamine-tetraacetic-acid (EDTA) and subsequently assayed ectoine synthase activity. The addition of very low concentrations of EDTA (0.05 mM) to the EctC enzyme already led to a noticeable inhibition of the ectoine synthase activity and the presence of 1 mM EDTA completely inhibited the enzyme (Fig 3a).

We then took such an inactivated enzyme preparation, removed the EDTA by dialysis, and added stoichiometric amounts (10 μM) of various metals to the (*Sa*)EctC enzyme. The addition of FeCl_2_ to the enzyme assay restored enzyme activity to about 38%, whereas the addition of ZnCl_2_ or CoCl_2_ rescued (*Sa*)EctC enzyme activity only to 5% and 3%, respectively. All other tested metals, including Fe^3+^, were unable to restore activity (Fig 3b). When the concentration of the various metals in the enzyme assay was increased 100-fold, Fe^2+^ exhibited again the strongest stimulating effect on enzyme activity, and rescued enzyme activity to a degree similar to that exhibited by (*Sa*)EctC protein preparations that had not been inactivated through EDTA treatment (Fig 3c). However, a large molar excess of other transition-state metals (zinc, cobalt, nickel, copper, and manganese) typically found in members of the cupin superfamily [52–55] allowed the partial rescue of ectoine synthase activity as well (Fig 3c). This is in line with literature data showing that cupin-type enzymes are often promiscuous with respect to the use of the catalytically important metal [56–58].

### Kinetic parameters of EctC for *N*-γ-ADABA and *N*-α-ADABA

Based on the data presented in S3 Fig, we formulated an optimized activity assay for the ectoine synthase of *S*. *alaskensis* and used it to determined the kinetic parameters for the (*Sa*)EctC enzyme for both its natural substrate *N*-γ-ADABA [21, 35] and the isomer *N*-α-ADABA. The EctC-catalyzed ring-closure of *N*-γ-ADABA to form ectoine exhibited Michaelis-Menten-kinetics with an apparent *K*_m_ of 4.9 ± 0.5 mM, a *v*_max_ of 25.0 ± 0.8 U/mg and a *k*_cat_ of 7.2 s^-1^ (S4a Fig). Given the chemical relatedness of *N*-α-ADABA to the natural substrate (*N*-γ-ADABA) of the ectoine synthase (S1a and S1b Fig), we wondered whether (*Sa*)EctC could also use *N*-α-ADABA to produce ectoine. This was indeed the case. (*Sa*)EctC catalyzed this reaction with Michaelis-Menten-kinetics exhibiting an apparent *K*_m_ of 25.4 ± 2.9 mM, a *v*_max_ of 24.6 ± 1.0 U/mg and a *k*_cat_ 0.6 s^-1^ (S4b Fig). Hence, *N*-α-ADABA is a newly recognized substrate for ectoine synthase. However, both the affinity (*K*_m_) of the (*Sa*)EctC protein and its catalytic efficiency (*k*_cat_/*K*_m_) were strongly reduced in comparison with *N*-γ-ADABA. The *K*_m_ dropped fife-fold from 4.9 ± 0.5 mM to 25.4 ± 2.9 mM, and the catalytic efficiency was reduced from 1.47 mM^-1^ s^-1^ to 0.02 mM^-1^ s^-1^, a 73-fold decrease.

Both *N*-γ-ADABA and *N*-α-ADABA are concomitantly formed during the enzymatic hydrolysis of the ectoine ring during catabolism [51]. Our finding that *N*-α-ADABA is a substrate for ectoine synthase has bearings for an understanding of the physiology of those microorganisms that can both synthesize and catabolize ectoine [51]. However, these types of microorganisms should still be able to largely avoid a futile cycle since the affinity of ectoine synthase for *N*-γ-ADABA and *N*-α-ADABA, and its catalytic efficiency for the two compounds, differs substantially (S4a and S4b Fig).

### Crystallization of the (*Sa*)EctC protein

Since no crystal structure of ectoine synthase has been reported, we set out to crystallize the (*Sa*)EctC protein. Attempts to obtain crystals of (*Sa*)EctC in complex either with its substrate *N*-γ-ADABA or its reaction product ectoine were not successful. However, two crystal forms of the (*Sa*)EctC protein in the absence of the substrate were obtained. Crystal form A diffracted to 1.2 Å and had a unit cell of a = 72.71 b = 72.71 c = 52.33 Å and α = 90 β = 90 γ = 120° displaying a *P*3_2_21 symmetry (S1 Table). Crystal form B diffracted to 2.0 Å and had a unit cell of a = 97.52 b = 43.96 c = 138.54 Å and α = 90 β = 101.5 γ = 120° and displayed a *C*2 symmetry (S1 Table). Attempts to solve the crystal structure of the (*Sa*)EctC protein by molecular replacement has previously failed [41]. However, we were able to obtain crystals of form B that were derivatized with mercury and these diffracted up to 2.8 Å (S1 Table). This dataset was used to derive an initial structural model of the (*Sa*)EctC protein, which in turn was employed as a template for molecular replacement to phase the native dataset (2.0 Å) of crystal form B. After several rounds of manual model building and refinement, four monomers of (*Sa*)EctC were identified and the crystal structure was refined to a final R_cryst_ of 21.1% and an R_free_ of 24.8% (S1 Table). Finally, a monomer of this structure was used as a template for molecular replacement to phase the high-resolution (1.2 Å) dataset of crystal form A, which was subsequently refined to a final R_cryst_ of 12.4% and an R_free_ of 14.9% (S1 Table).

### Overall fold of the (*Sa*)EctC protein

**Fig 4:**
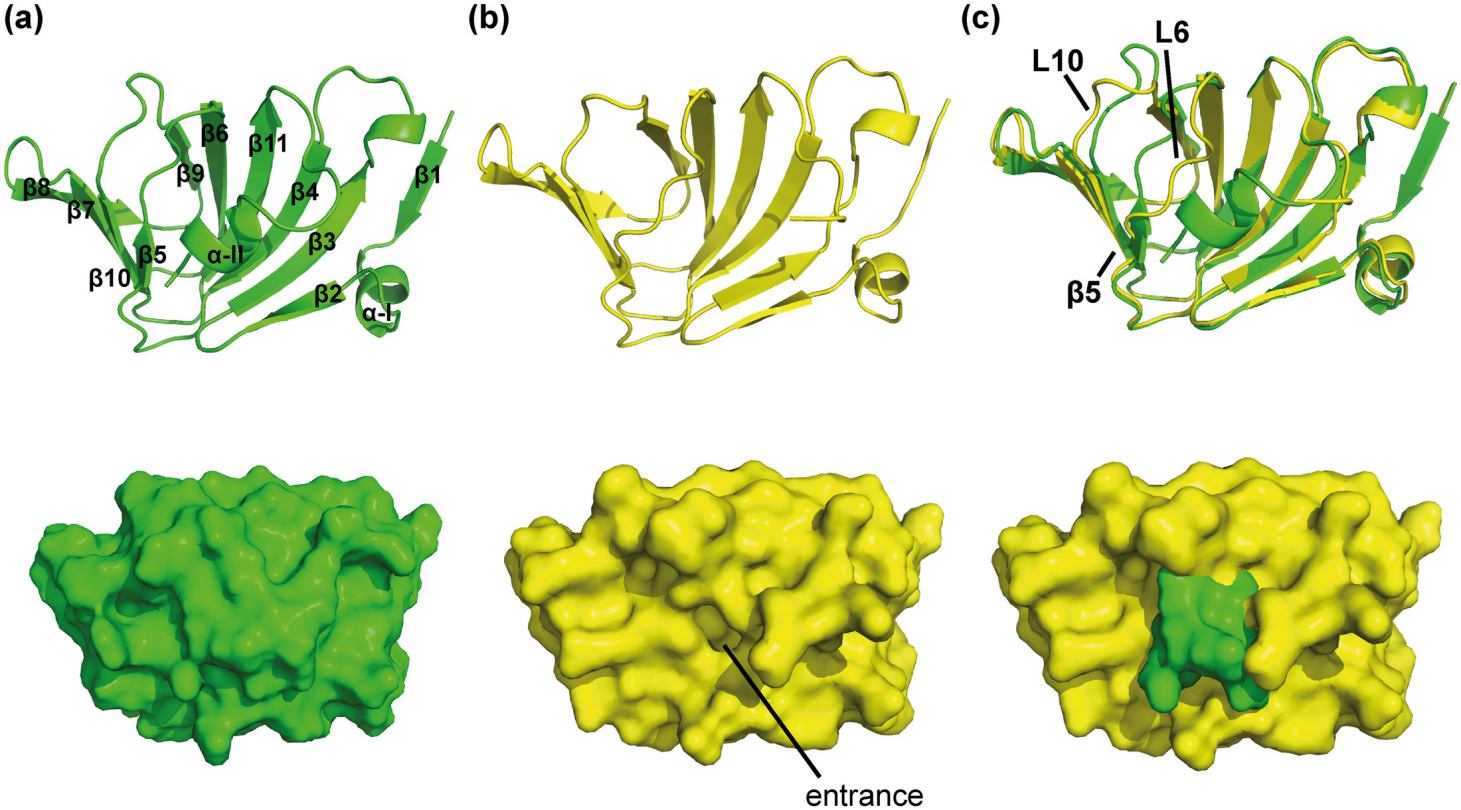
Overall structure of the “open” and “semi-closed” crystal structures of (*Sa*)EctC. (a) The overall structure of the “semi-closed” (*Sa*)EctC resolved at 2.0 Å is depicted in green in a cartoon (upper panel) and surface (lower panel) representation. The β-strands are numbered β1-β11 and the helices α-I to α-II. (b) The overall structure of the “open” (*Sa*)EctC was resolved at 1.2 Å and is depicted in yellow in a cartoon (upper panel) and surface (lower panel) representation. The entrance to the active site of the ectoine synthase is marked. (c) Overlay of the “semi-closed” and “open” (*Sa*)EctC structures.

The two EctC structures that we determined revealed that the ectoine synthase belongs to the cupin superfamily [52–55] with respect to its overall fold (Fig 4a–4c). However, they represent two different states of the 137 amino acids comprising (*Sa*)EctC protein (Fig 2). First, the 1.2 Å structure reveals the spatial configuration of the (*Sa*)EctC protein ranging from amino acid Met-1 to Glu-115; hence, it lacks 22 amino acids at the carboxy-terminus of the authentic (*Sa*)EctC protein. This structure adopts an open conformation with respect to the typical fold of cupin barrels [52–55] and is therefore termed in the following the “open” (*Sa*)EctC structure (Fig 4b). In this structure no metal co-factor was identified. The second crystal structure of the (*Sa*)EctC protein was solved at a resolution of 2.0 Å and contained four molecules of the protein in the asymmetric unit of which protomer A comprised amino acid Met-1 to Gly-121 and adopts a closed conformation. Hence, it still lacks 16 amino acid residues of the carboxy-terminus of the authentic 137 amino acids comprising (*Sa*)EctC protein (Fig 2). We therefore cannot exclude that this crystal structure does not represent the fully closed state of the ectoine synthase; consequently, we tentatively termed it the “semi-closed” (*Sa*)EctC structure. Interestingly, the three other monomers present in the asymmetric unit all range from Met-1 to Glu-115 and adopt a conformation similar to the “open” EctC structure.

The overall structure of (*Sa*)EctC is basically the same in both crystals except for the carboxy-terminus, which covers the entry of one side of the cupin barrel from the surroundings in monomer A in the “semi-closed” structure. This is reflected by the calculated root mean square deviation (RMSD) of the Cα atoms that was about 0.56 Å (over 117 residues) when the four “open” monomers were compared with each other. However, the “semi-closed” monomer has a slightly higher RMSD of 1.4 Å (over 117 residues) when compared with the “open” 2.0 Å structure. Therefore, we describe in the following the overall structure for the “semi-closed” form of the (*Sa*)EctC protein and subsequently highlight the structural differences between the “open” and “semi-closed” forms in more detail.

The structure of the “semi-closed” (*Sa*)EctC protein consists of 11 β-strands (β1-β11) and two α-helices (α-I and α-II) (Fig 4a). The β-strands form two anti-parallel β-sheets: β2 β3, β4, β11, β6, and β9, and a smaller three-stranded β-sheet (β7, β8, and β10), respectively. These two β-sheets pack against each other, forming a cup-shaped β-sandwich with a topology characteristic for the cupin-fold [52–55]. Hence, (*Sa*)EctC adopts an overall bowl shape in which one side is opened towards the solvent (Fig 4a to 4c). In the “semi-closed” structure, a longer carboxy-terminal tail is visible in the electron density, folding into a small helix (α-II) that closes the active site of the (*Sa*)EctC protein (Fig 4a). The formation of this α-II helix induces a reorientation and shift of a long unstructured loop (as observed in the “open” structure) connecting β4 and β6, resulting in the formation of the stable β-strand β5 as observed in the “semi-closed”state of the (*Sa*)EctC protein (Fig 4a).

Structural comparison analyses using the DALI server [59] revealed that (*Sa*)EctC adopts a fold similar to other members of the cupin superfamily [52–55]. The highest structural similarities are observed for the Cupin 2 conserved barrel domain protein (YP_751781.1) from *Shewanella frigidimarina* (PDB accession code: 2PFW) with a Z-score of 13.1 and an RMSD of 2.2 Å over 104 Cα-atoms (structural data for this protein have been deposited in the PDB but no publication connected to this structure is currently available), a manganese-containing cupin (TM1459) from *Thermotoga maritima* (PDB accession code: 1VJ2) with a Z-score of 12.8 and an RMSD of 2.0 Å over 103 Cα-atoms [60], the cyclase RemF from *Streptomyces resistomycificus* (PDB accession code: 3HT1 with a Z-score of 11.9 and an RMSD of 1.9 Å over 102 Cα-atoms) [56], and an auxin-binding protein 1 from *Zea mays* (PDB accession code: 1LR5) with an Z-score of 11.8 and an RMSD of 2.8 Å over 104 Cα-atoms) [61]. Our data classify EctC, in addition to the polyketide cyclase RemF [56], as the second known cupin-related enzyme that catalyze a cyclocondensation reaction. Next to RemF [56] and the aldos-2-ulose dehydratase/isomerase [62], the ectoine synthase is only the third characterized dehydratase within the cupin superfamily.

### Analysis of the EctC dimer interface as observed in the (*Sa*)EctC crystal structure

**Fig 5:**
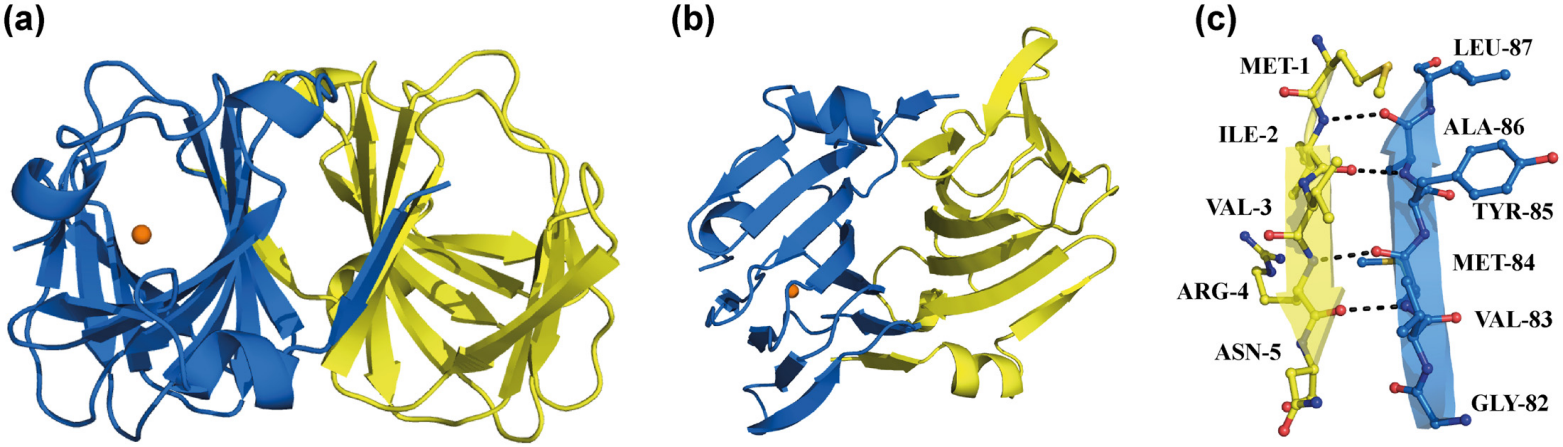
Crystal structure of (*Sa*)EctC. (a) Top-view of the dimer of the (*Sa*)EctC protein. The position of the water molecule, described in detail in the text, is shown in one of the monomers as an orange sphere. (b) Side-view of a (*Sa*)EctC dimer allowing an assessment of the dimer interface formed by two β-strands of each monomer. (c) Close-up representation of the dimer interface mediated by beta-strand β1 and β6.

Both the SEC analysis [41] and the HPLC-MALS experiments (S2b Fig) have shown that the ectoine synthase from *S*. *alaskensis* is a dimer in solution. The crystal structure of this protein reflects this quaternary arrangement. In the “semi-closed” crystal structure, (*Sa*)EctC has crystallized as a dimer of dimers within the asymmetric unit. This dimer (Fig 5a and 5b) is composed of two monomers arranged in a head-to-tail orientation and is stabilized via strong interactions mediated by two antiparallel β-strands, β-strand β1 (sequence ^1^MIVRN^5^) from monomer A and β-strand β8 from monomer B (sequence ^82^GVMYAL^87^) (Fig 5c). The strong interactions between these β-strands rely primarily on backbone contacts. In addition to these interactions, some weaker hydrophobic interactions are also observed between the two monomers in some loops connecting the β-strands. As calculated with PDBePISA [63], the surface area buried upon dimer formation is 1462 Å^2^, which is 20.5% of the total accessible surface of a monomer of this protein. Both values fall within the range for known functional dimers.

In the “open” (*Sa*)EctC structure, one monomer is present in the asymmetric unit. We therefore inspected the crystal packing and analyzed the monomer-monomer interactions with symmetry related molecules to elucidate whether a physiologically relevant dimer could be deduced from this crystal form as well. Indeed, a similar dimer configuration to the one described for the “semi-closed” (Sa)EctC structure is observed with the same monomer-monomer interactions mediated by the two β-sheets. The crystallographic two-fold axis present within the crystal symmetry is located exactly in between the two monomers, resulting in a monomer within the asymmetric unit. Hence, the same dimer observed in the “semi-closed” structure of (*Sa*)EctC can also be observed in the “open” structure. Interestingly, the proteins identified by the above-described DALI search not only have folds similar to EctC, but are also functional dimers that adopt similar monomer-monomer interactions within the dimer assembly as deduced from the inspection of the corresponding PDB files (2PFW, 3HT1, 1VJ2, 1LR5).

### Structural rearrangements of the flexible (*Sa*)EctC carboxy-terminus

The cupin core [52–55] represents the structural framework of ectoine synthase (Figs 4 and 5). The major difference in the two crystal structures of the (*Sa*)EctC protein reported here is the orientation of the carboxy-terminus. Some amino acids located in the carboxy-terminal region of the 137 amino acids comprising (*Sa*)EctC protein are highly conserved (Fig 2) within the extended EctC protein family [13, 14]. At the end of β-strand β11, two consecutive conserved proline residues (Pro-109 and Pro-110) are present that are responsible for a turn in the main chain of the (*Sa*)EctC protein. In the “semi-closed” (*Sa*)EctC structure, the visible electron density of the carboxy-terminus is extended by 7 amino acid residues and ends at position Gly-121. These additional amino acids fold into a small helix, which seals the open cavity of the cupin-fold of the (*Sa*)EctC protein (Fig 4a). Furthermore, this helix is stabilized via interactions with the loop region between β-strands β4 and β6, thereby inducing a structural rearrangement. This induces the formation of β-strand β5, which is not present when the small C-terminal helix is absent as observed in the “open” (*Sa*)EctC structure. As a result, the newly formed β-strand β5 is reoriented and moved by 2.4 Å within the “semi-closed” (*Sa*)EctC structure (Fig 4a to 4c). It is worth mentioning that β-strand β5 is located next to His-93, which in all likelihood involved in metal binding (see below). The position of this His residue is slightly shifted in both (*Sa*)EctC structures, likely the result of the formation of β-strand β5. Therefore the sealing of the cupin fold, as described above, seem to have an indirect influence on the architecture of the postulated iron-binding site.

The consecutive Pro-109 and Pro-110 residues found at the end of β-strand β11are highly conserved in EctC-type proteins (Fig 2) [13, 14]. They are responsible for redirecting the main chain of the remaining carboxy-terminus (27 amino acid residues) of (*Sa*)EctC to close the cupin fold. In the “semi-closed” structure this results in a complete closure of the entry of the cupin barrel (Fig 4a to 4c). In the “open” (*Sa*)EctC structure, both proline residues are visible in the electron density; however, almost directly after Pro-110, the electron density is drastically diminished caused by the flexibility of the carboxy-terminus. A search for partners interacting with Pro-109 revealed that it interacts via its backbone oxygen with the side chain of His-55 as visible in both the “open” and “semi-closed” (*Sa*)EctC structures. The Pro-109/His-55 interaction ensures the stable orientation of both proline residues at the end of β-strand β11. Since these proline residues are followed by the carboxy-terminal region of the (*Sa*)EctC protein, the interaction of His-55 with Pro-109 will likely play a substantial role in spatially orienting this very flexible part of the protein.

**Fig 6:**
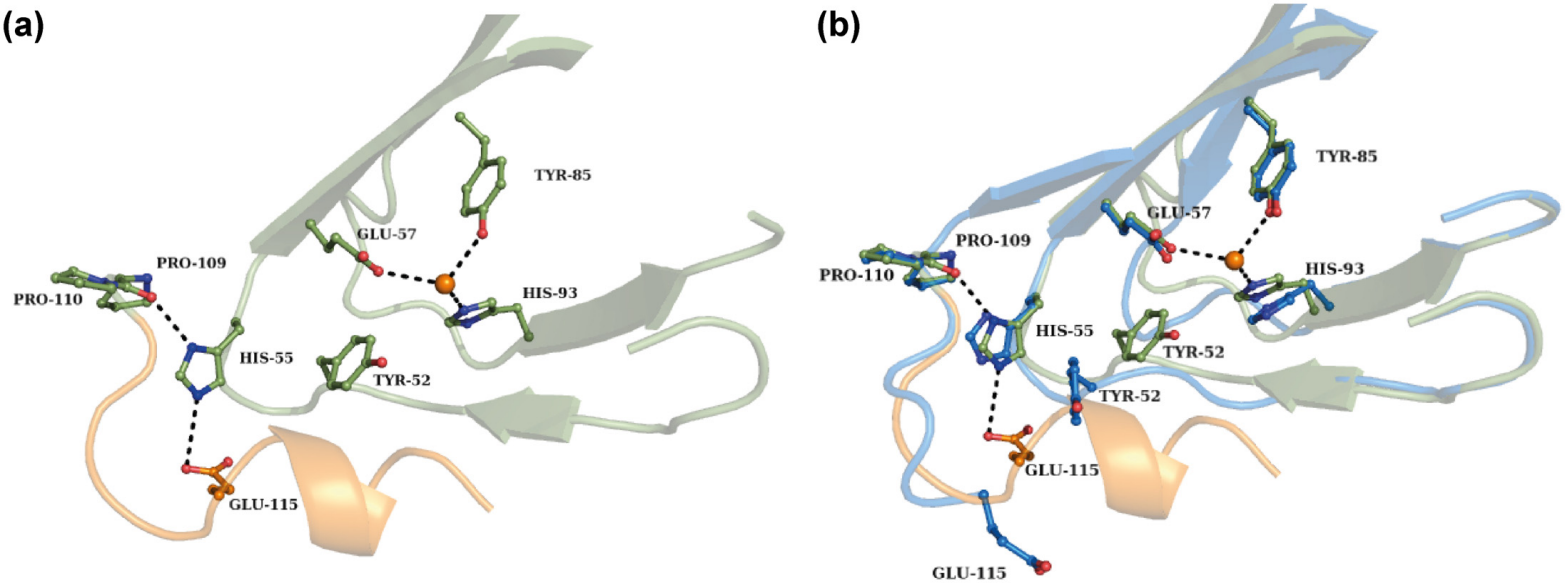
Architecture of the presumed metal-binding site of the (*Sa*)EctC protein and its flexible carboxy-terminus. (a) The described water molecule (depicted as orange sphere) is bound via interactions with the side chains of Glu-57, Tyr-85, and His-93. The position occupied by this water molecule represents probably the position of the Fe^2+^ cofactor in the active side of the ectoine synthase. His-55 interacts with the double proline motif (Pro-109 and Pro-110). It is further stabilized via an interaction with the side chain of Glu-115 which is localized in the flexible carboxy-terminus (colored in orange) of (*Sa*)EctC that is visible in the “semi-closed” (*Sa*)EctC structure. (b) An overlay of the “open” (colored in light blue) and the “semi-closed” (colored in green) structure of the (*Sa*)EctC protein.

In addition to the interactions between Pro-109 and His-55, the carboxy-terminal region of (*Sa*)EctC is held in position via an interaction of Glu-115 with His-55, which stabilizes the conformation of the small helix in the carboxy-terminus further. The interaction between Glu-115 and His-55 is only visible in the “semi-closed” structure where the partially extended carboxy-terminus is resolved in the electron density. In the “open” structure of the (*Sa*)EctC protein, this interaction does not occur since Glu-115 is rotated outwards (Fig 6a and 6b). Hence, one might speculate that this missing interaction might be responsible for the flexibility of the carboxy-terminus in the “open” (*Sa*)EctC structure and consequently results in less well defined electron density in this region.

### The putative iron binding site of (*Sa*)EctC

In the “semi-closed” structure of (*Sa*)EctC, each of the four monomers in the asymmetric unit contains a relative strong electron density positioned within the cupin barrel. Since (*Sa*)EctC is a metal containing protein (Fig 3), we tried to fit either Fe^2+^, or Zn^2+^ ions into this density and also refined occupancy. Only the refinement of Fe^2+^ resulted in a visibly improved electron density, however with a low degree of occupancy. This possible iron molecule is bound via interactions with Glu-57, Tyr-85 and His-93 (Fig 6a and 6b). The distance between the side chains of these residues and the (putative) iron co-factor is 3.1 Å for Glu-57, 2.9 Å for Tyr-85, and 2.9 Å for His-93, respectively. These distances are to long when compared to other iron binding sites, a fact that might be caused by the absence of the proper substrate in the (*Sa*)EctC crystal structure. Since both the refinement and the distance did not clearly identify an iron molecule, we decided to conservatively place a water molecule at this position. The position of this water molecule is described in more detail below and is highlighted in Figs 5a and 5b and 6a and 6b as a sphere. Interestingly, all three amino acids coordinating this water molecule are strictly conserved within an alignment of 440 members of the EctC protein family [13] (for an abbreviated alignment of EctC-type proteins see Fig 2).

In the “open” structure of the (*Sa*)EctC protein, electron density is visible where the presumptive iron is positioned in the “semi-closed” structure. However, this electron density fits perfectly to a water molecule and not to an iron, and the water molecule was clearly visible after the refinement at this high resolution (1.2 Å) of the “open” (*Sa*)EctC structure. In a superimposition of both (*Sa*)EctC crystal structures, the spatial arrangements of the side chains of the three amino acids (Glu-57, Tyr-85, and His-93) likely to contact the iron in the “semi-closed” structure match nicely with those of the corresponding residues of the “iron-free” “open” structure (Fig 6b). Only His-93 is slightly rotated inwards in the “semi-closed” structure, most likely due to formation of β-strand β5 as described above. Taken together, this observations indicate, that the architecture of the presumptive iron-binding site is pre-set for the binding of the catalytically important metal by the ectoine synthase.

Of note is the different spatial arrangement of the side-chain of Tyr-52 (located in a loop after the end of β-strand β5) in the “open” and “semi-closed” (*Sa*)EctC structures. In the “semi-closed” structure, the hydroxyl-group of the side-chain of Tyr-52 points towards the iron (Fig 6a and 6b), but the corresponding distance (3.9 Å) makes it highly unlikely that Tyr-52 is directly involved in metal binding. Nevertheless, its substitution by an Ala residue causes a strong decrease in iron-content and enzyme activity of the mutant protein (Table 1). It becomes apparent from an overlay of the “open” and “semi-closed” (*Sa*)EctC crystal structures that the side-chain of Tyr-52 rotates away from the position of the presumptive iron, whereas the side-chains of those residues that probably contacting the metal directly [Glu-57, Tyr-85, and His-93], remain in place (Fig 6a and 6b). Since Tyr-52 is strictly conserved in an alignment of 440 EctC-type proteins [13] (Fig 2), we speculate that it might be involved in contacting the substrate of the ectoine synthase and that the absence of *N*-γ-ADABA in our (*Sa*)EctC crystal structures might endow the side chain of Tyr-52 with extra spatial flexibility.

To further analyze the putative iron binding site (Fig 6a), we performed structure-guided site-directed mutagenesis and assessed the resulting (*Sa*)EctC variants for their iron content [43] and studied their enzyme activity. When those three residues (Glu-57, Tyr-85, His-93) that likely form the mono-nuclear iron center in the (*Sa*)EctC crystal structure were individually replaced by an Ala residue, both the catalytic activity and the iron content of the mutant proteins was strongly reduced (Table 1). For some of the presumptive iron-coordinating residues, additional site-directed mutagenesis experiments were carried out. To verify the importance of the negative charge in the position of Glu-57, we created an Asp variant. This mutant protein rescued the enzyme activity and iron content of the Ala substitution substantially (Table 1). We also replaced Tyr-85 with either a Phe or a Trp residue and both mutant proteins largely lost their catalytic activity and iron content (Table 1) despite the fact that these substitutions were conservative. Collectively, these data suggest that the hydroxyl group of the Tyr-85 side chain is needed for the binding of the iron (Fig 6a). We also replaced the presumptive iron-binding residue His-93 by an Asn residue, yielding a (*Sa*)EctC protein variant that possessed an enzyme activity of 23% and iron content of only 14% relative to that of the wild-type protein (Table 1). Collectively, the data addressing the functionality of the putative iron-coordinating residues (Glu-57, Tyr-85, His-93) buttress our notion that the Fe^2+^ present in the (*Sa*)EctC protein is of catalytic importance.

### A chemically undefined ligand in the (*Sa*)EctC structure provides clues for the binding of the *N*-γ-ADABA substrate

Despite considerable efforts, either by trying co-crystallization or soaking experiments, we were not able to obtain a (*Sa*)EctC crystal structures that contained either the substrate *N*-γ-ADABA, or ectoine, the reaction product of ectoine synthase (Fig 1). However, in the “semi-closed” (*Sa*)EctC structure where the carboxy-terminal loop is largely resolved, a long stretched electron density feature was detected in the predicted active site of the enzyme; it remained visible after crystallographic refinement. This is in contrast to the high-resolution “open” structure of the (*Sa*)EctC protein where no additional electron density was observed after refinement.

We tried to fit all compounds used in the buffers during purification and crystallization into the observed electron density, but none matched. This observation indicates that the chemically undefined ligand was either trapped by the (*Sa*)EctC protein during its heterologous production in *E*. *coli* or during crystallization. Since we used PEG molecules in the crystallization conditions, the observed density might stem from an ordered part of a PEG molecule, or low molecular weight PEG species that might have been present in the PEG preparation used in our experiments. We therefore stress that we cannot identify neither the true chemically nature of this compound, nor its precise origin.

**Fig 7:**
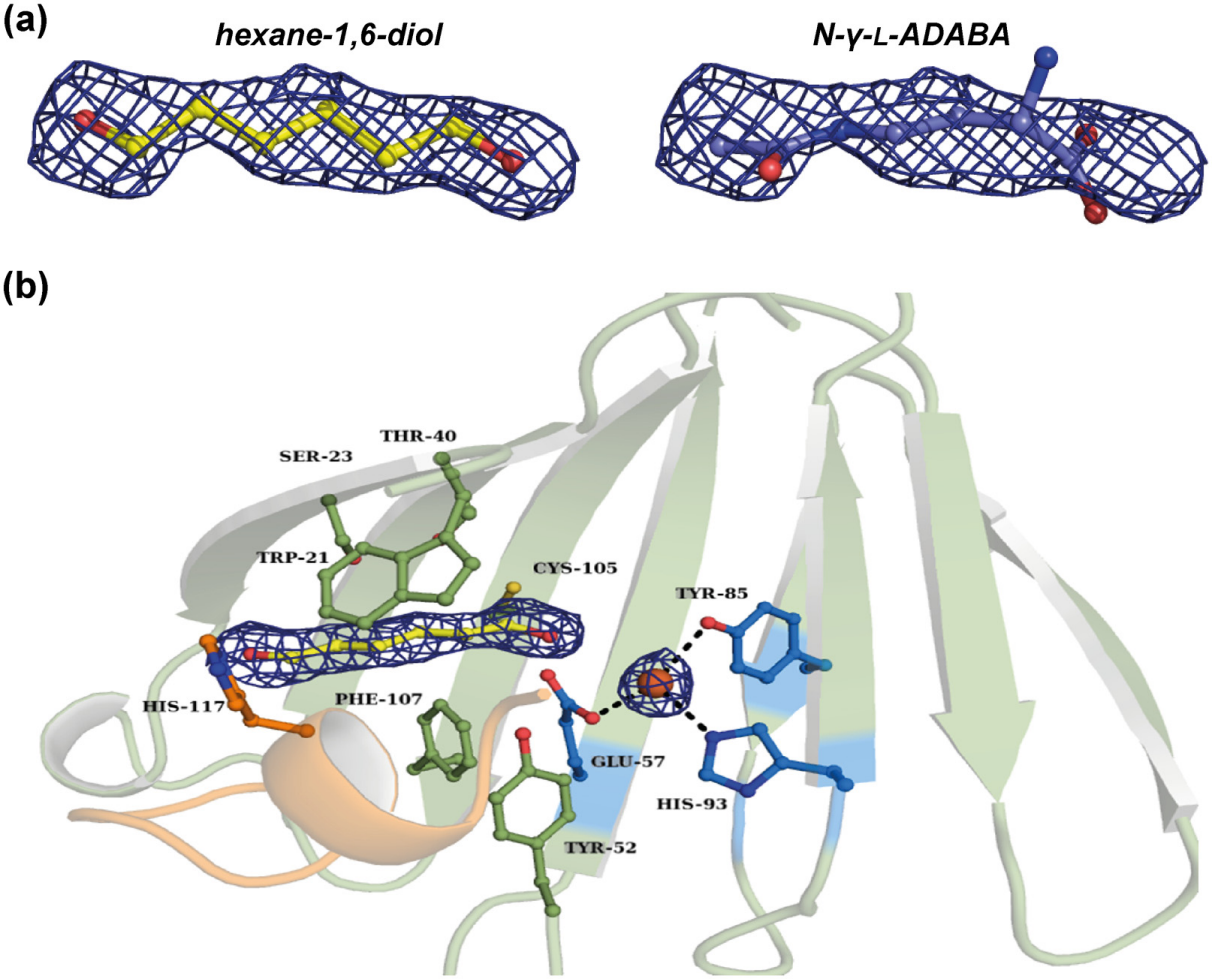
A chemically undefined ligand is captured in the active site of the “semi-closed” (*Sa*)EctC crystal structure. (a) The observed electron density in the active site of the “semi-closed” structure of (*Sa*)EctC is modeled as a hexane-1,6-diol molecule and compared with the electron density of the *N*-γ-ADABA substrate of the ectoine synthase to emphasize the similarity in size of these compounds. (b) The presumable binding site of the iron co-factor and of the modeled hexane-1,6-diol molecule is depicted. The amino acid side chains involved in iron-ligand binding are colored in blue and those involved in the binding of the chemically undefined ligand are colored in green using a ball and stick representation. The flexible carboxy-terminal loop of (*Sa*)EctC is highlighted in orange. The electron density was calculated as an omit map and contoured at 1.0 σ.

Estimating from the dimensions of the electron density feature, we modeled the chemically undefined compound trapped by the (*Sa*)EctC protein as a hexane-1,6-diol molecule (PDB identifier: HEZ) to best fit the observed electron density. However, to the best of our knowledge, hexane-1,6-diol is not part of the *E*. *coli* metabolome [64]. Despite these notable limitations, we considered the serendipitously trapped compound as a mock ligand that might provide useful insights into the spatial positioning of the true EctC substrate and those residues that coordinate it within the ectoine synthase active site. We note that both *N*-γ-ADABA and hexane-1,6-diol are both C6-compounds and display similar length (Fig 7a).

We refined the (*Sa*)EctC structure with the trapped compound, and by doing so, the refinement parameters (especially R- and R_free_-factor) dropped by 1.5%. We also calculated an omit map and the electron density reappeared (Fig 7b). When analyzing the interactions of this compound within the (*Sa*)EctC protein, we found that it is bound via interactions with Trp-21 and Ser-23 of β-sheet β3, Thr-40 located in β-sheet β4, and Cys-105 and Phe-107, which are both part of β-sheet β11. Remarkably, all of these residues are highly conserved throughout the extended EctC protein family (Fig 2) [13, 14].

### Structure-guided site-directed mutagenesis of the catalytic core of the ectoine synthase

In a previous alignment of the amino acid sequences of 440 EctC-type proteins, 13 amino acids were identified as strictly conserved residues [13]. These correspond to amino acids Thr-40, Tyr-52, His-55, Glu-57, Gly-64, Tyr-85- Leu-87, His-93, Phe-107, Pro-109, Gly-113, Glu-115, and His-117 in the (*Sa*)EctC protein (Fig 2). Amino acid residues Gly-64, Pro-109, and Gly-113 likely fulfill structural roles since they are positioned either at the end or at the beginning of β-strands and α-helices. We considered the remaining ten residues as important either for ligand binding, for catalysis, or for the structurally correct orientation of the flexible carboxy-terminus of the (*Sa*)EctC protein. As described above, the side chains of Glu-57, Tyr-85, and His-93 are probably involved in iron binding (Table 1 and Fig 6a).

In view of the (*Sa*)EctC structure with the serendipitously trapped compound (Fig 7b), we probed the functional importance of the seven residues that contact this ligand by structure-guided site-directed mutagenesis (Table 1). Each of these mutant (*Sa*)EctC proteins was overproduced in *E*. *coli* and purified by affinity chromatography; they all yielded pure and stable protein preparations. We benchmarked the activity of the (*Sa*)EctC variants in a single time-point enzyme assay under conditions where 10 μM of the wild-type (*Sa*)EctC protein converted almost completely the supplied 10 mM *N*-γ-ADABA substrate to 9.33 mM ectoine within a time frame of 20 min. In addition, we determined the iron content of each of the mutant (*Sa*)EctC protein by a colorimetric assay [43] (Table 1).

The side chains of the evolutionarily conserved Trp-21, Ser-23, Thr-40, Cys-105, and Phe-107 residues (Fig 2) make contacts with the chemically undefined ligand that we observed in the “semi-closed” (*Sa*)EctC structure (Fig 7b). We replaced each of these residues with an Ala residue and found that none of them had an influence on the iron content of the mutant proteins. However, their catalytic activity was substantially impaired (Table 1). Thr-40 is positioned on β-strand β5 and its side chain protrudes into the lumen of the cupin barrel formed by the (*Sa*)EctC protein (Fig 7b). We also replaced Phe-107 with either an Tyr or an Trp residue: the Phe-107/Tyr substitution possessed near wild-type enzyme activity (about 95%) and the full iron content, but the Phe-107/Trp substitution possessed only 12% enzyme activity and 72% iron content compared to the wild-type protein. The properties of these mutant proteins indicate that the aromatic side chain at position 107 of (*Sa*)EctC is of importance but that a substitution with a bulky aromatic side chain is strongly detrimental to enzyme activity and concomitantly moderately impairs iron binding. Replacement of the only Cys residue in (*Sa*)EctC (Cys-105; Fig 2) by a Ser residue, a configuration that is naturally found in two EctC proteins among 440 inspected amino acid sequences [13], yielded a (*Sa*)EctC variant with 84% wild-type activity and an iron content similar to that of the wild-type protein. However, the Cys-105/Ala variant was practically catalytically inactive while largely maintaining its iron content (Table 1). Since the side-chains of Cys residues are chemically reactive and often participate in enzyme catalysis, Cys-105 (or Ser-105) might serve such a role for ectoine synthase.

We observed two amino acid substitutions that simultaneously strongly affected enzyme activity and iron content; these were the Tyr-52/Ala and the His-55/Ala (*Sa*)EctC protein variants (Table 1). Based on the (*Sa*)EctC crystal structures that we present here, we can currently not firmly understand why the replacement of Tyr-52 by Ala impairs enzyme function and iron content so drastically (Table 1). This is different for the His-55/Ala substitution. The carboxy-terminal region of the (*Sa*)EctC protein is held in its position via an interaction of Glu-115 with His-55, where His-55 in turn interacts with Pro-110 (Fig 6a and 6b). Each of these residues is evolutionarily highly conserved [13]. The individual substitution of either Glu-115 or His-55 by an Ala residue is predicted to disrupt this interactive network and therefore should affect enzyme activity. Indeed, the Glu-115/Ala and the His-55/Ala substitutions possessed only 21% and 16% activity of the wild-type protein, respectively (Table 1). The Glu-115/Ala mutant possessed wild-type levels of iron, whereas the iron content of the His-55/Ala substitutions dropped to 15% of the wild-type level (Table 1). We also replaced Glu-115 with a negatively charged residue (Asp); this (*Sa*)EctC variant possessed wild-type levels of iron and still exhibited 77% of wild-type enzyme activity. Collectively, these data suggest that the correct positioning of the carboxy-terminus of the (*Sa*)EctC protein is of structural and functional importance for the activity of the ectoine synthase.

Residues Leu-87 and Asp-91 are highly conserved in the ectoine synthase protein family [13, 14]. The replacement of Leu-87 by Ala led to a substantial drop in enzyme activity (Table 1). Conversely, the replacement of Asp-91 by Ala and Glu, resulted in (*Sa*)EctC protein variants with 80% and 98% enzyme activity, respectively (Table 1). We currently cannot comment on possible functional role Asp-91. However, Leu-87 is positioned at the end of one of the β-sheets that form the dimer interface (Fig 5c) and it might therefore possess a structural role. It is also located near Tyr-85, one of the residues that probably coordinate the iron molecule with in the (*Sa*)EctC active site (Fig 6a) and therefore might exert indirect effects. His-117 is a strictly conserved residue and its substitution by an Ala residue results in a drop of enzyme activity (down to 44%) and an iron content of 83% (Table 1). We note that His-117 is located close to the chemically undefined ligand in the (*Sa*)EctC structure (Fig 7b) and might thus play a role in contacting the natural substrate of the ectoine synthase.

As an internal control for our mutagenesis experiments, we also substituted Thr-41 and His-51, two residues that are not evolutionarily conserved in EctC-type proteins [13, 14] with Ala residues. Both (*Sa*)EctC protein variants exhibited wild-type level enzyme activities and possessed a iron content matching that of the wild-type (Table 1). This illustrates that not every amino acid substitution in the (*Sa*)EctC protein leads to an indiscriminate impairment of enzyme function and iron content.

## Discussion

The crystallographic data presented here firmly identify ectoine synthase (EctC), an enzyme critical for the production of the microbial cytoprotectant and chemical chaperone ectoine [12, 14, 20, 33], as a new member of the cupin superfamily. The overall fold and bowl shape of the (*Sa*)EctC protein (Figs 4 and 5) with its 11 β-strands (β1-β11) and two α-helices (α-I and α-II) closely adheres to the design principles typically found in crystal structures of cupins [52–55]. In addition to the ectoine synthase, the polyketide cyclase RemF is the only other currently known cupin-related enzyme that catalyze a cyclocondensation reaction [56] although the substrates of EctC and RemF are rather different. As a consequence of the structural relatedness of EctC and RemF and the type of chemical reaction these two enzymes catalyze, is now understandable why *bona fide* EctC-type proteins are frequently (mis)-annotated in microbial genome sequences as “RemF-like” proteins.

The pro- and eukaryotic members of the cupin superfamily perform a variety of both enzymatic and non-enzymatic functions that are built upon a common structural scaffold [53, 55]. Most cupins contain transition state metals that can promote different types of chemical reactions [52, 54]. Except for some cupin-related proteins that seem to function as metallo-chaperones [65], the bound metal is typically an essential part of the active sites [55, 56, 66, 67]. We report here for the first time that the ectoine synthase is a metal-dependent enzyme. ICP-MS, metal-depletion and reconstitution experiments (Fig 3) consistently identify iron as the biologically most relevant metal for the EctC-catalyzed cyclocondensation reaction. However, as observed with other cupins [56–58], EctC is a somewhat promiscuous enzyme as far as the catalytically important metal is concerned when they are provided in large molar excess (Fig 3c).

Although some uncertainty remains with respect to the precise identity of amino acid residues that participate in metal binding by (*Sa*)EctC, our structure-guided site-directed mutagenesis experiments targeting the presumptive iron-binding residues (Fig 6a and 6b) demonstrate that none of them can be spared (Table 1). The architecture of the metal center of ectoine synthase seems to be subjected to considerable evolutionary constraints. The three residues (Glu-57, Tyr-85, His-93) that we deem to form it (Figs 6 and 7b) are strictly conserved in a large collection of EctC-type proteins originating from 16 bacterial and three archaeal phyla (Fig 2) [13, 14].

We also show here for the first time that, in addition to its natural substrate *N*-γ-ADABA, EctC also converts the isomer *N*-α-ADABA into ectoine, albeit with a 73-fold reduced catalytic efficiency (S3a and S3b Fig). Hence, the active site of ectoine synthase must possess a certain degree of structural plasticity, a notion that is supported by the report on the EctC-catalyzed formation of the synthetic compatible solute ADPC through the cyclic condensation of two glutamine molecules [36]. Our finding that *N*-α-ADABA serves as a substrate for ectoine synthase has physiologically relevant ramifications for those microorganisms that can both synthesize and catabolize ectoine [51], since they need to prevent a futile cycle of synthesis and degradation when *N*-α-ADABA is produced as an intermediate in the catabolic route.

Although we cannot identify the true chemical nature of the C6 compound that was trapped in the (*Sa*)EctC structure nor its precise origin, we treated this compound as a proxy for the natural substrate of ectoine synthase, which is a C6 compound as well (Fig 7a). We assumed that its location and mode of binding gives, in all likelihood, clues as to the position of the true substrate *N*-γ-ADABA within the EctC active site. Indeed, site-directed mutagenesis of those five residues that contact the unknown C6 compound (Fig 7b) yielded (*Sa*)EctC variants with strongly impaired enzyme function but near wild-type levels of iron (Table 1). This set of data and the fact that the targeted residues are strongly conserved among EctC-type proteins (Fig 2) [13, 14] is consistent with their potential role in *N*-γ-ADABA binding or enzyme catalysis. We therefore surmise that our crystallographic data and the site-directed mutagenesis study reported here provide a structural and functional view into the architecture of the EctC active site (Fig 7b).

The ectoine synthase from the cold-adapted marine bacterium *S*. *alaskensis* [40] can be considered as a psychrophilic enzyme (S3a Fig), types of proteins with a considerable structural flexibility [68, 69]. This probably worked to the detriment of our efforts in solving crystal structures of the full-length (*Sa*)EctC protein in complex with either *N*-γ-ADABA or ectoine. Because microbial ectoine producers can colonize ecological niches with rather different physicochemical attributes, it seems promising to exploit this considerable biodiversity [13, 14] to identify EctC proteins with enhanced protein stability. It is hoped that these can be further employed to obtain EctC crystal structures with either the substrate or the reaction product. Together with our finding that ectoine synthase is metal dependent, these crystal structures should allow a more detailed understanding of the chemistry underlying the EctC-catalyzed cyclocondensation reaction.

## Supporting Information

S1 Fig ^1^H- and ^13^C-NMR spectroscopy of synthesized *N*-γ-ADABA and *N*-α-ADABA. The purity and identity of (a) *N*-γ-ADABA and (b) *N*-α-ADABA was assessed by both ^1^H-NMR and ^13^C-NMR spectroscopy as described [39, 70].
(TIF)

S2 Fig Purity of the isolated (*Sa*)EctC and analysis of its oligomeric state by HPLC-MALS. (a) The (*Sa*)EctC-Strep-tag II protein was purified by affinity chromatography and its purity was analyzed on an 15% SDS-polyacrylamide gel. The PageRuler Prestained Protein Ladder was used as a marker to assess the electrophoretic mobility of the (*Sa*)EctC protein. (b) The oligomeric state of the purified (*Sa*)EctC was determined by high-performance liquid chromatography coupled to multi-angle light scattering detection (HPLC-MALS) analysis. The black line reflects the normalized refractive index detector signal and the black dotted line represents the calculated protein mass.
(TIF)

S3 Fig Basic biochemical parameters of (*Sa*)EctC. The enzyme activity of the purified (*Sa*)EctC protein is shown with respect to (a) its temperature, (b) its pH profile and the influence of sodium chloride and potassium chloride is depicted in (c) and (d), respectively.
(TIF)

S4 Fig Kinetic parameters of the (*Sa*)EctC enzyme for its substrates *N*-γ-ADABA and *N*-α-ADABA. Michealis-Menten-kinetics of the purified (*Sa*)EctC protein for (a) its natural substrate *N*-γ-ADABA and (b) the isomer *N*-α-ADABA.
(TIF)

S1 Table Data collection and refinement statistics for structural analysis of the (*Sa*)EctC protein. (DOCX)
