# Supplementary figures and images for: Biochemistry and Crystal Structure of Ectoine Synthase: A Metal-Containing Member of the Cupin Superfamily
Source: PLoS One. 2016 Mar 17;11(3):e0151285. doi: 10.1371/journal.pone.0151285 (PMC4795551; doi:10.1371/journal.pone.0151285)

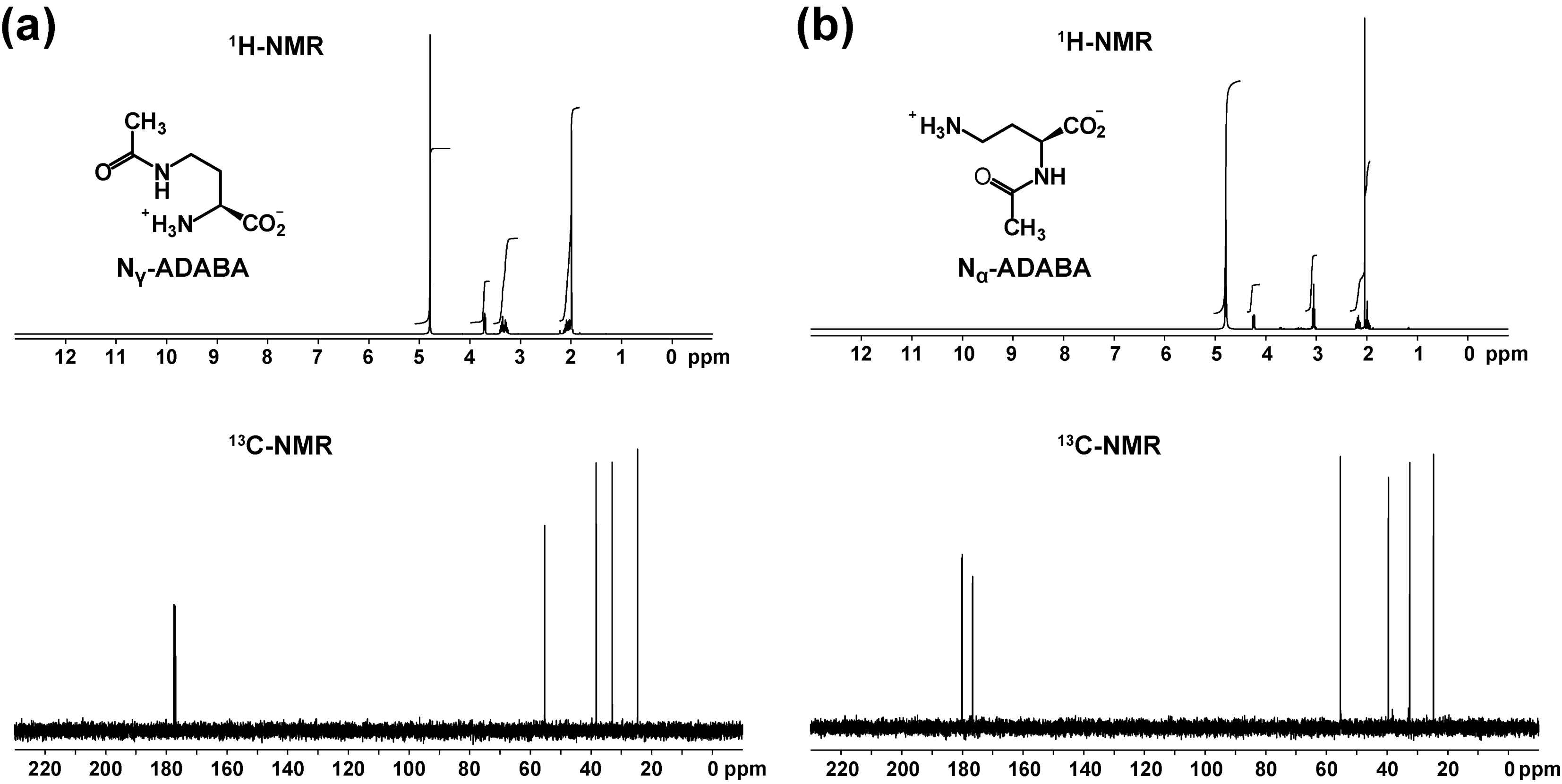

Supplement: S1 Fig — The purity and identity of (a) N-γ-ADABA and (b) N-α-ADABA was assessed by both 1H-NMR and 13C-NMR spectroscopy as described [39, 70]. (TIF) [file pone.0151285.s001.tif]

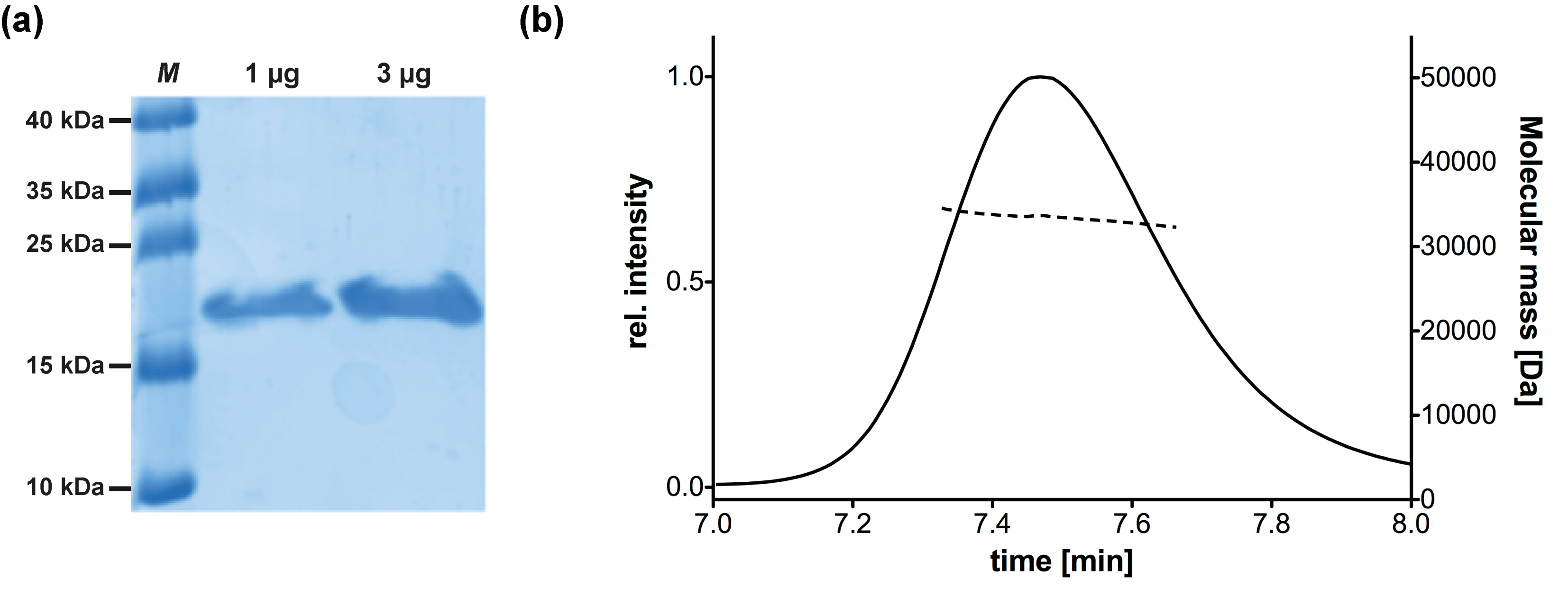

Supplement: S2 Fig — (a) The (Sa)EctC-Strep-tag II protein was purified by affinity chromatography and its purity was analyzed on an 15% SDS-polyacrylamide gel. The PageRuler Prestained Protein Ladder was used as a marker to assess the electrophoretic mobility of the (Sa)EctC protein. (b) The oligomeric state of the purified (Sa)EctC was determined by high-performance liquid chromatography coupled to multi-angle light scattering detection (HPLC-MALS) analysis. The black line reflects the normalized refractive index detector signal and the black dotted line represents the calculated protein mass. (TIF) [file pone.0151285.s002.tif]

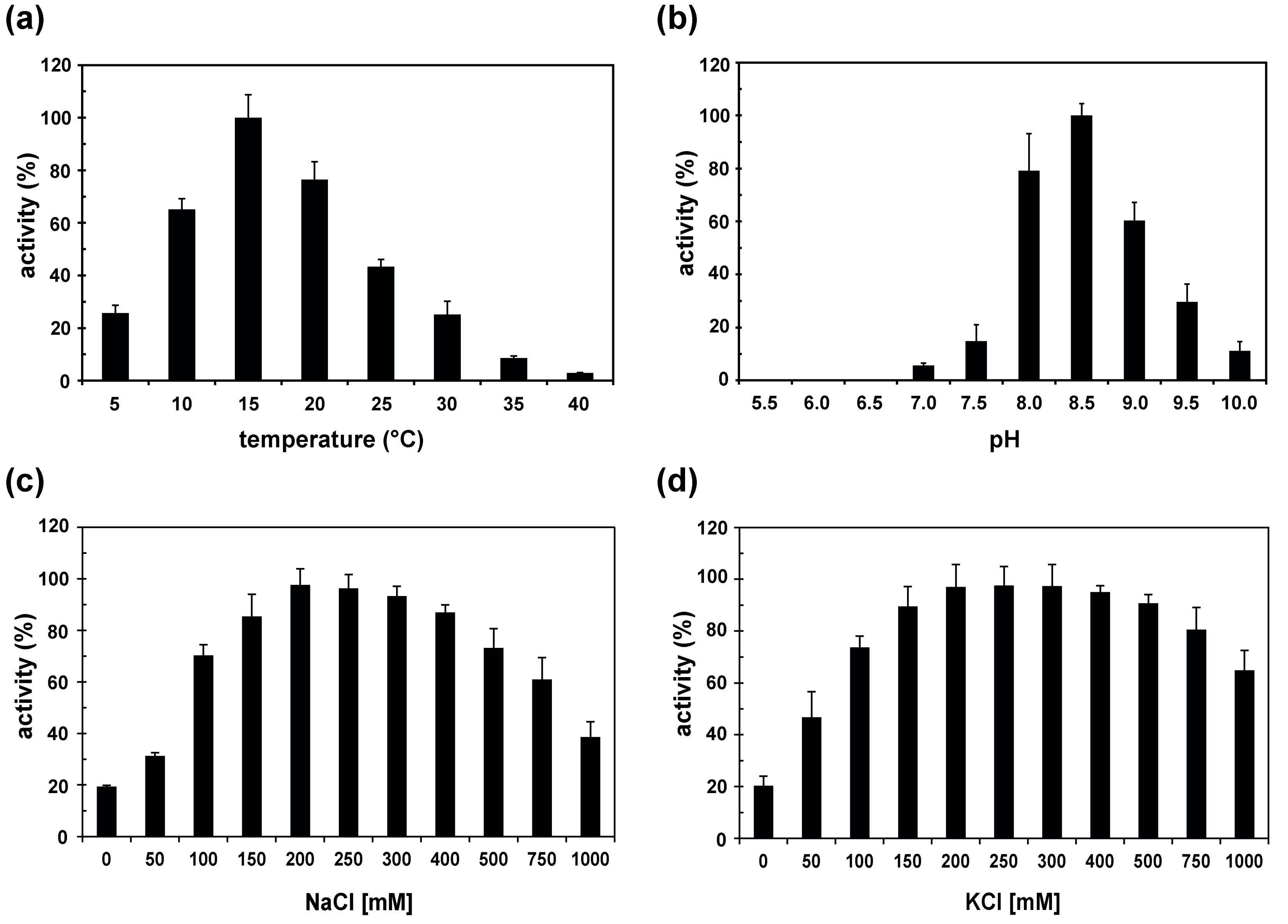

Supplement: S3 Fig — The enzyme activity of the purified (Sa)EctC protein is shown with respect to (a) its temperature, (b) its pH profile and the influence of sodium chloride and potassium chloride is depicted in (c) and (d), respectively. (TIF) [file pone.0151285.s003.tif]

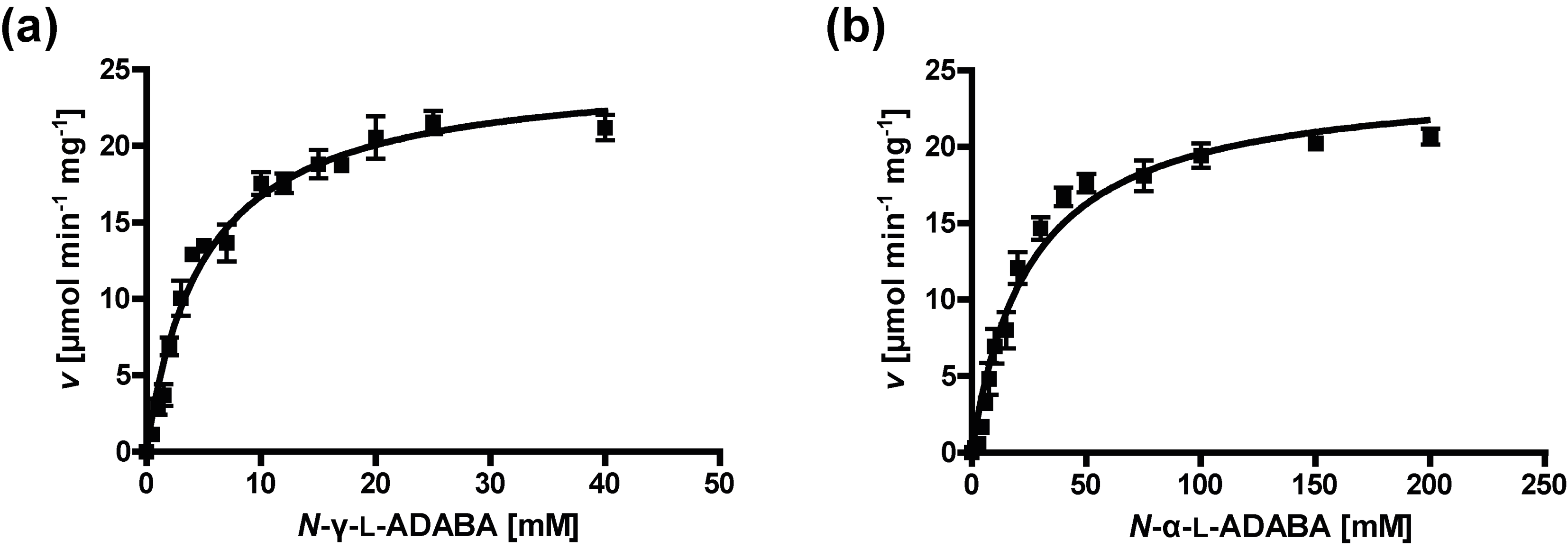

Supplement: S4 Fig — Michealis-Menten-kinetics of the purified (Sa)EctC protein for (a) its natural substrate N-γ-ADABA and (b) the isomer N-α-ADABA. (TIF) [file pone.0151285.s004.tif]
