# Supplementary material for: Biochemistry and Crystal Structure of Ectoine Synthase: A Metal-Containing Member of the Cupin Superfamily
Source: PLoS One. 2016 Mar 17;11(3):e0151285. doi: 10.1371/journal.pone.0151285 (PMC4795551; doi:10.1371/journal.pone.0151285)
Supplement: S1 Table — (DOCX) [file pone.0151285.s005.docx]

**S1 Table. Data collection and refinement statistics.**

|  | **„Open“**  ***Sa*EctC** | **„Semi-closed“**  ***Sa*EctC** | **Mercury-**  ***Sa*EctC** |
| --- | --- | --- | --- |
| *Data collection* |  |  |  |
| Wavelength (Å) | 0.97625 | 0.97625 | 0.97625 |
| Space group | *P* 3_2_ 2 1 | *C* 1 2 1 | *C* 1 2 1 |
| Cell dimensions  a, b, c (Å)  α, β, γ (°) | 72.71 72.71 52.33  90.0 90.0 120.0 | 97.52 43.96 138.54  90.0 101.5 90.0 | 97.47 43.95 138.68  90.0 101.98 90.0 |
| Resolution (Å) | 19.4-1.2 (1.3-1.2) ^a^ | 45.2-2.0 (2.1-2.0) ^a^ | 45.2-2.8 (2.9-2.8) ^a^ |
| *R*_merge_ (%) ^b^ | 6.6 (12.2) | 9.2 (53.2) | 8.2 (14.1) |
| *I/σI* | 21.9 (14.1) | 11.8 (3.0) | 16.3 (10.6) |
| Completeness (%) | 97.8 (94.7) | 98.73 (98.9) | 99.0 (99.5) |
| Redundancy | 7.6 (5.2) | 5.7 (4.2) | 6.2 (6.8) |
|  |  |  |  |
| Total reflections | 728378 (75037) | 221513 (16191) | 213593 (14514) |
| No. of unique reflections | 49094 (4701) | 38862 (3855) | 14369 (2650) |
| Molecules per asymmetric unit | 1 | 4 | 4 |
|  |  |  |  |
| *Refinement* | | | |
| Resolution (Å) | 19.4-1.2 (1.3-1.2) | 45.2-2.0 (2.1-2.0) |  |
| No. of reflections | 48490 | 36918 | 14369 |
| *R*_work_/*R*_free_ (%) | 12.1/15.2 | 20.9/25.7 |  |
| No. of atoms |  |  |  |
| Protein | 954 | 7093 |  |
| Ligand | 34 | 20 |  |
| Iron | - | 4 |  |
| Water | 110 | 201 |  |
| Average B-factor (A^2^) | 17.70 | 27.70 |  |
| macromolecules | 15.40 | 27.90 |  |
| Ligand | 30.60 | 23.50 |  |
| Water | 111 | 196 |  |
| Protein residues | 1 Monomer | 4 Monomers |  |
|  | | | |
| Root mean-square deviations | | | |
| Bond lengths (Å) | 0.019 | 0.008 |  |
| Bond angles (°) | 2.19 | 1.20 |  |
| Ramachandran plot | | | |
| Favored regions (%) | 100 | 97.1 |  |
| Allowed regions (%) | 0 | 2.9 |  |
| Outliers (%) | 0 | 0 |  |
| ^a^ Values for the highest-resolution shell are shown in parenthesis.  ^b^ *R*_merge_ = ΣhklΣ_i_ \|I_i_(hkl) − <I(hkl)>\|/ΣhklΣ_i_I_i_(hkl), where I_i_(hkl) is the intensity of the *i*th observation of reflection hkl and <I(hkl)> is the average over all observations of reflection hkl. ^c^ *R*_factor_ = Σhkl‖F_o_\| − \|F_c_‖/Σhkl\|F_o_\| for all data excluding the 5% that comprised the *R*_free_ used for cross-validation. | | | |
